# Suppression of NNK Metabolism by Anthocyanin-Rich Haskap Berry Supplementation Through Modulation of P450 Enzymes

**DOI:** 10.3390/ph17121615

**Published:** 2024-11-30

**Authors:** Madumani Amararathna, David W. Hoskin, Kerry B. Goralski, H. P. Vasantha Rupasinghe

**Affiliations:** 1 Department of Plant, Food, and Environmental Sciences, Faculty of Agriculture, Dalhousie University, Truro, NS B2N 5E3, Canada; madu.ama@dal.ca; 2 Department of Pathology, Faculty of Medicine, Dalhousie University, Halifax, NS B3H 4H7, Canada; d.w.hoskin@dal.ca; 3 Department of Pharmacology, Faculty of Medicine, Dalhousie University, Halifax, NS B3H 4H7, Canada; kerry.goralski@dal.ca; 4 Department of Pediatrics, Faculty of Medicine, Dalhousie University, Halifax, NS B3H 4H7, Canada; 5 College of Pharmacy, Dalhousie University, Halifax, NS B3H 4R2, Canada; 6 Division of Hematology/Oncology, IWK Health Centre, Halifax, NS B3K 6R8, Canada; 7 Beatrice Hunter Cancer Research Institute, Halifax, NS B3H 4R2, Canada

**Keywords:** blue honeysuckle, cytochrome P450, hepatic metabolism, carcinogen, lung cancer, smoking

## Abstract

Oral supplementation of anthocyanins-rich haskap (*Lonicera caerulea*) berry (HB) reduces 4-(methylnitrosamino)-1-(3-pyridyl)-1-butanone (NNK)-induced lung tumorigenesis, cytotoxicity, DNA damage, and modulated inflammation in vitro and in vivo. The procarcinogen NNK is metabolically activated by cytochrome P450 (P450) enzymes, producing reactive metabolites that induce lung carcinogenesis. **Hypothesis**: Therefore, we hypothesized that the HB-modulated protective effect against NNK could be due to its ability to suppress P450 enzymes. **Methods**: HB (6 mg of cyanidin-3-*O*-glucoside [C3G] in 0.2 g of HB/mouse/day) was given to A/J mice as a dietary supplement following subsequent administration of NNK (100 mg/kg body weight). The liver tissues of mice were analyzed to determine the expression of P450s and metabolites. **Results**: HB upregulated the expression of *cyp2a4* and *cyp2a5* mRNA and nuclear receptor/transcription factor (PPARα) in NNK-deprived hepatic tissues. With NNK, HB downregulated the expression of *cyp2a4* and *cyp2a5* and facilitated the formation of non-carcinogenic NNK metabolites. Molecular docking indicated a high binding affinity and strong hydrophobic interactions between C3G and its major metabolites, peonidin-3-*O*-glucoside, petunidin-3-*O*-glucoside, peonidin and cyanidin with Cyp2a5 and with human P450 homologue CYP2A13. **Conclusions**: HB could be a potential dietary supplement to inhibit the P450 activated NNK carcinogenic metabolites formation. Hence, inhibiting the activation of NNK by lung CYP2A13 through dietary HB supplementation could be a strategy to reduce lung carcinogenesis among smokers. Understanding the effect of HB on the activity of CYP2A13 in human studies is necessary before recommending these natural compounds as therapeutics.

## 1. Introduction

**Figure 1:**
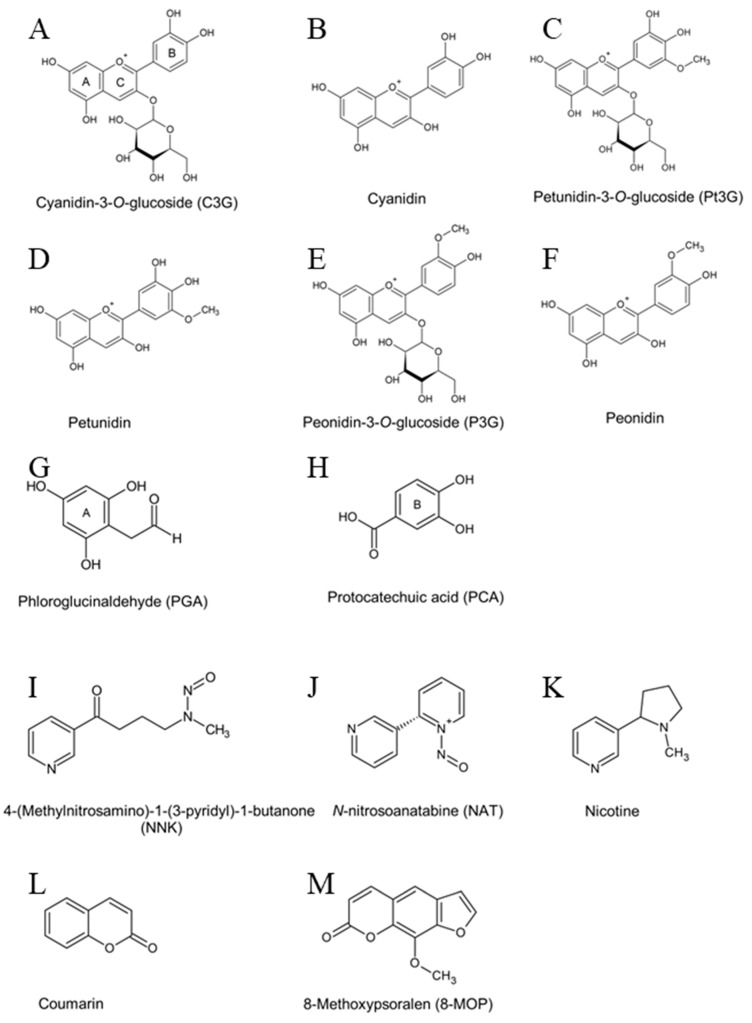
The chemical structures of haskap berry anthocyanins and their metabolites (**A**–**H**), tobacco-specific carcinogens (**I**–**K**), a common ligand (**L**), and an inhibitor (**M**) of cytochrome 450 enzymes. A, A-ring, B, B-ring, and C, C-ring of the C3G structure; a, the methyl carbon atom of NNK; b, the methylene carbon atom of NNK.

The cytochrome P450 (P450) superfamily enzymes govern the metabolism of xenobiotics, including carcinogens such as nicotine and 4-(methylnitrosamino)-1-(3-pyridyl)-1-butanone (NNK). NNK is a substrate of the CYP2A subfamily enzymes [1]. As phase I enzymes, P450 oxidizes, reduces, and hydrolyzes diverse ligands by incorporating polar groups into its substrates. Its structural plasticity allows conformational shifts, enabling the enzyme to bind nonpolar molecules and nitrogen-containing compounds. The active site supports different binding modes: Type I, where hydrophobic interactions displace water, and Type II, where nitrogen-containing ligands directly coordinate with the heme iron. This flexibility enables CYP2A to metabolize a broad range of substrates efficiently [2,3]. CYP2A6 and CYP2A13 in humans, Cyp2a4 and Cyp2a5 in mice, and Cyp2a3 in rats catalyze the hydroxylation of methyl and methylene sites adjacent to the *N*-nitroso group of NNK. The resulting electrophilic metabolites can react with DNA to generate DNA adducts, leading to lung cancer [4,5,6]. Genetic polymorphisms in CYP2A13, but not CYP2A6, have been associated with a threefold increase in lung cancer risk among women exposed to second-hand smoke in Indonesia [7]. In comparison, the oral intake of the P450 inhibitor, 8-methoxypsoralen (8-MOP) (Figure 1), suppresses the formation of electrophilic metabolites of NNK and promotes the generation of non-genotoxic glucuronides in smokers [5]. Similarly, certain dietary molecules, such as coumarin-rich celery extracts along with flavonoids (kaempferol and myricetin), have been identified for their inhibitory effects on Cyp2a5, CYP2A6, and CYP2A13 [4,8]. Thus, molecularly targeting CYP-specific procarcinogen activation in lung and liver tissues with CYP-inhibiting dietary molecules could be a novel approach to reducing the carcinogen-derived electrophilic metabolites and subsequent genotoxicity while driving NNK biotransformation to non-toxic metabolites that are excreted from the body without causing harm.

Haskap (*Lonicera caerulea*) berry (HB) is a rich source of flavonoids, particularly cyanidin-3-*O*-glucoside (C3G), a type of anthocyanin. Our prior research has confirmed the ability of HB to prevent or mitigate NNK-induced cytotoxicity and lung tumorigenesis in vitro and in vivo [9,10]. However, the mechanisms responsible for this protective effect have not been elucidated. Based on the CYP-inhibiting properties of other flavonoids, we hypothesized that HB anthocyanins inhibit NNK-metabolizing P450 enzymes leading to reduced carcinogenic metabolite production; therefore, reduced lung tumorigenesis. Additionally, HB anthocyanins modulate the expression of pro-inflammatory cytokines IL-6, tumor necrosis factor-alpha (TNF-α), prostaglandin E_2_, and the cyclooxygenase-2 enzyme [11]. It is also reported that CYP2A13 in the lungs can be suppressed by carcinogen-induced pro-inflammatory cytokines, including IL-6 and TNF-α [12]. For instance, lipopolysaccharide treatment reduces *cyp2a5* mRNA (46%), protein (35%) and activity (23%) after 24 h in DBA/2N mouse livers [13]. Thus, the secondary hypothesis is that the modulation of inflammatory pathways by HB anthocyanins indirectly suppresses the expression of P450 enzymes, reducing the hepatic activation of carcinogenicity and toxicity of NNK. The objectives of the present study were to (1) evaluate the effect of HB on the expression of P450 enzymes in the liver of NNK-challenged mice, (2) determine the immunomodulatory effect of HB in NNK-exposed mice, and (3) study the ligand binding affinities and interactions of primary anthocyanin metabolites of HB with CYP2A13, CYP2A6 by using molecular docking analysis.

## 2. Results

### 2.1. HB Supplementation Suppressed the Expression of CYP2A Enzymes in NNK-Challenged Mouse Hepatic Tissues

**Figure 2:**
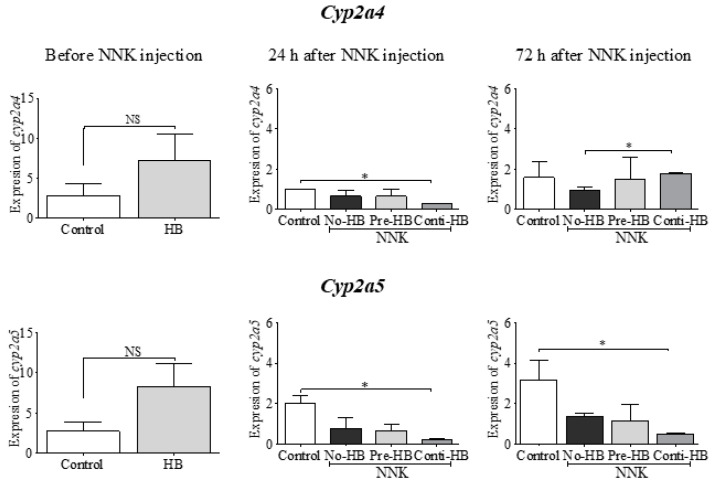
The effect of HB and NNK on the expression of hepatic *cyp* mRNA. Hepatic *cyp2a4* and *cyp2a5* mRNA in HB-fed A/J mice (6 mg of C3G in 0.2 g of HB/mouse/day for 21 days) compared to regular chow-fed A/J mice before (left column), 24 h (middle column) and 72 h (right column) after NNK (100 mg/kg body weight, i.p.) treatment. Pre-HB mice received HB before NNK, Conti-HB mice received HB before and after NNK, No-HB mice did not receive HB, and the controls were administered an equal volume of saline i.p. Each data set is expressed as mean ± SD. * The indicated groups were significantly different (*p* < 0.05). One-way ANOVA followed by Tukey’s pairwise comparison. NS = not significantly different. HB, haskap berry; NNK, 4-(methylnitrosamino)-1-(3-pyridyl)-1-butanone.

A significant reduction in the expression of *cyp2a4* and *cyp2a5* mRNA was observed after NNK (24 h) exposure, and the continuation of the HB-rich diet following NNK (Conti-HB) further expedited the inhibition of these enzymes (Figure 2). Specifically, *cyp2a4* expression was reduced by 4-fold (*p* < 0.05) in the Conti-HB group compared to the control group. A parallel observation was noted for *cyp2a5* in NNK-challenged mice, where Conti-HB treatment led to a 10-fold downregulation (*p* < 0.05) compared to the control group. However, 72 h after NNK exposure, the expression of *cyp2a4* was restored to average levels in the NNK-injected mouse liver. On the contrary, *cyp2a5* remained significantly lower (*p* < 0.05) in the Conti-HB-fed mice compared to the control, even after the 72 h following the NNK exposure.

### 2.2. HB Supplementation Upregulated the Expression of Pro-Inflammatory Cytokines in A/J Mice

**Figure 3:**
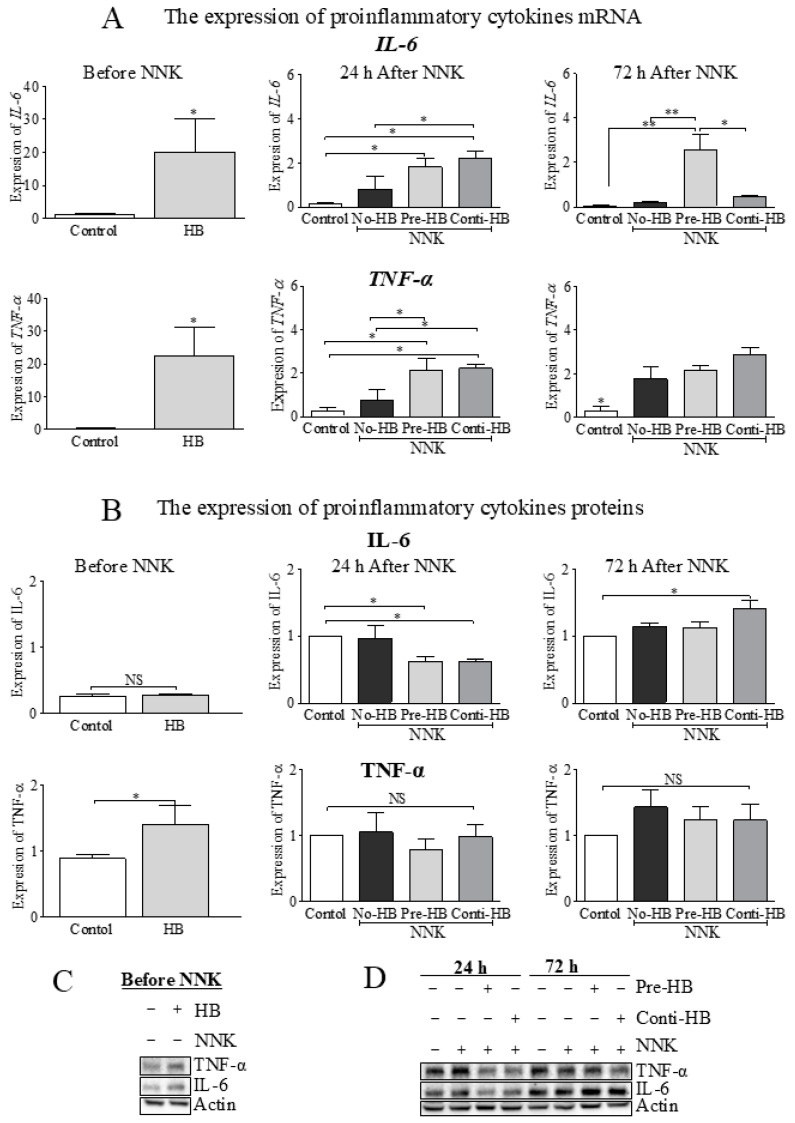
The effect of HB and NNK on the expression of pro-inflammatory cytokines in hepatic tissues of the A/J mice. (**A**) RT-PCR was performed to detect the expression of hepatic *IL-6* and *TNF-α* mRNA before (left column), 24 h (middle column) and 72 h (right column) after NNK injection. (**B**) Western blotting was conducted to measure the levels of IL-6 and TNF-α proteins before (left column) and after (middle and right columns) the NNK exposure. (**C**) The expression of proteins before NNK and (**D**) 24 h and 72 h after NNK injection. One-way ANOVA with the Bonferroni test (at α = 0.05) for mean comparison and *t*-test were performed. * and ** indicate statistical differences at *p* ≤ 0.05 and 0.01, respectively, with mean ± SD. NS = not significantly different. HB, haskap berry; NNK, 4-(methylnitrosamino)-1-(3-pyridyl)-1-butanone; Pre-HB mice received HB before NNK; Conti-HB mice received HB before and after NNK; No-HB mice did not receive HB, and the controls were administered an equal volume of saline i.p.

The expression of mRNA and proteins of hepatic cytokines were tested as illustrated (Figure 3). The pro-inflammatory cytokines *IL-6* and *TNF-α* (*p* < 0.05) mRNA were significantly upregulated among HB-fed (6 mg of C3G in 0.2 g HB/mouse/day for 21 days) mice than in the control (Figure 3A). NNK exposure did not significantly increase the *IL-6* or *TNF-α* levels after 24 h. However, the HB diet upregulated the *IL-6* and *TNF-α* (*p* < 0.05) in the NNK-injected mice compared to the control. In addition, *IL-6* expression was upregulated (*p* < 0.05) in the Conti-HB group, and *TNF-α* increased (*p* < 0.05) in Pre- and Conti-HB groups compared to No-HB. *IL-6* was downregulated following 72 h of NNK injection in No-HB and Conti-HB-fed mice. In contrast, the expression of *IL-6* remained constant in the Pre-HB (compared to 24 h) and significantly higher (*p* < 0.01) compared to the control and No-HB groups. After 72 h, *TNF-α* was significantly increased (*p* < 0.05) in NNK-challenged mouse liver tissues compared to the control group.

The expression of IL-6 and TNF-α proteins differed from their mRNA expression (Figure 3B–D). TNF-α was significantly increased (*p* < 0.05) after 21 days in HB-fed mice compared to the control. IL-6 decreased (*p* < 0.05) 24 h after NNK treatment in the HB-fed groups but significantly increased (*p* < 0.05) in the Conti-HB group compared to the control after 72 h. NNK or HB-dietary supplementation showed no significant effect on the TNF-α protein expression following 24 h and 72 h.

### 2.3. HB Supplementation Modulated the Energy Homeostasis in the Liver of A/J Mice

**Figure 4:**
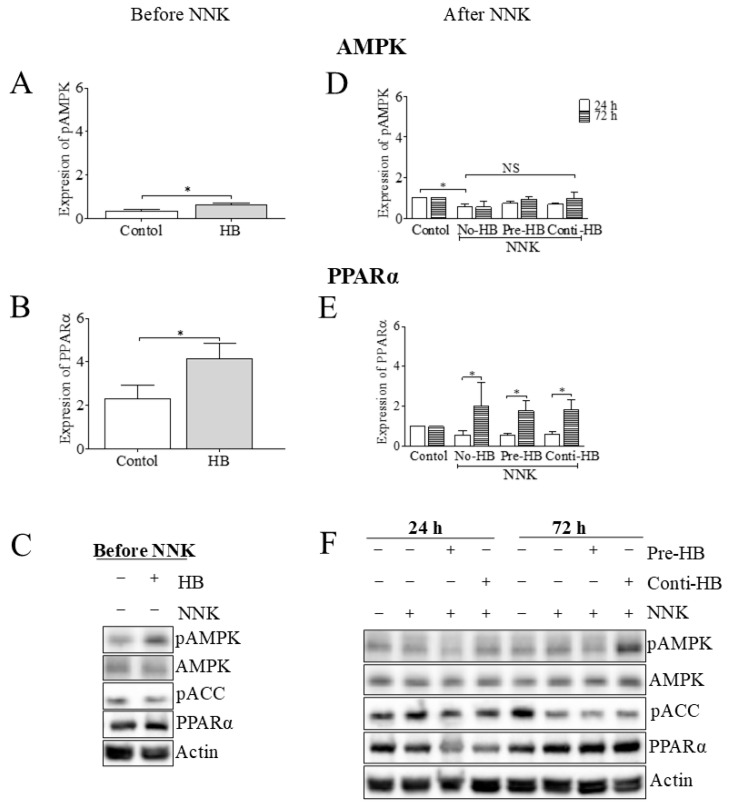
The effect of HB and NNK on lipogenic enzyme expression in hepatic tissues of the A/J mice. (**A**–**C**) Western blotting was conducted to measure phosphorylated AMPK and PPARα before NNK exposure, and (**D**–**F**) 24 h and 72 h after NNK exposure, respectively. Two-way ANOVA with the Bonferroni test (at α = 0.05) for mean comparison and *t*-test were performed. * Indicates statistical differences at *p* ≤ 0.05 with mean ± SD. NS = not significantly different. HB, haskap berry; NNK, 4-(methylnitrosamino)-1-(3-pyridyl)-1-butanone; Pre-HB mice received HB before NNK; Conti-HB mice received HB before and after NNK; No-HB mice did not receive HB, and the controls were administered an equal volume of saline i.p.

In healthy mice, the HB-supplemented diet increased the expression of AMP-activated protein kinase (AMPK) and peroxisome proliferator-activated receptor-alpha (PPARα) (*p* < 0.05) compared to the control, respectively (Figure 4A–C). The NNK or HB-rich diet did not significantly affect the expression of AMPK in NNK-challenged mouse liver. Conversely, PPARα was significantly induced (*p* < 0.05) in NNK-challenged groups after 72 h compared to that of the 24 h exposure (Figure 4D–F).

### 2.4. The Bioavailability of Anthocyanins and NNK Metabolites

**Figure 5:**
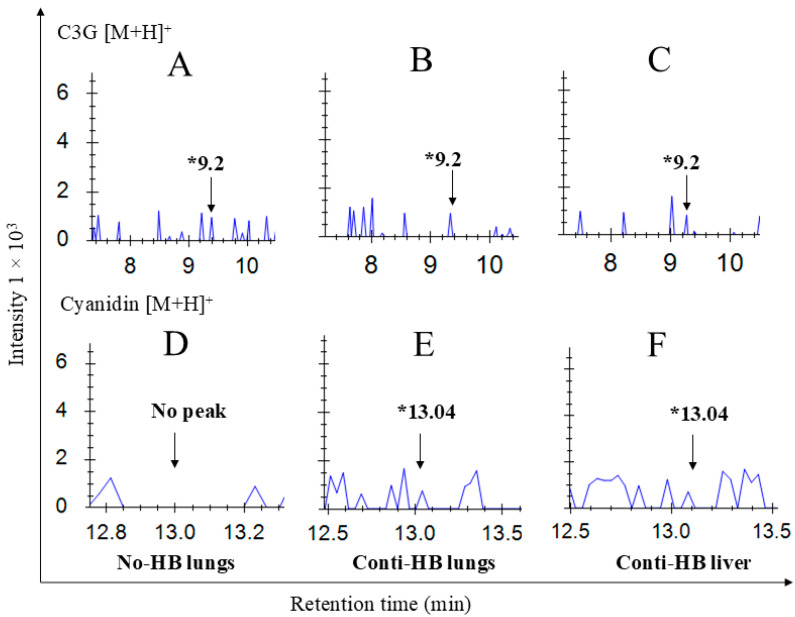
Detection of cyanidin-3-*O*-glucoside (C3G) and its primary metabolites present in lung and liver tissues of A/J mice. Lung and liver tissues were collected following 24 h of NNK injection and analyzed by UPLC/ESI/Q-TOF/MS for anthocyanin metabolites. C3G and cyanidin were detected in HB-fed mice tissues after 9.2 min and 13.04 min, respectively. Chromatograms shown are (**A**) lungs of mice from No-HB, (**B**) lungs of mice from Conti-HB, (**C**) liver of Conti-HB, and cyanidin of (**D**) No-HB, (**E**) lung of Conti-HB, and (**F**) liver of Conti-HB. HB, haskap berry; NNK, 4-(methylnitrosamino)-1-(3-pyridyl)-1-butanone; Conti-HB mice received HB before and after NNK; No-HB mice did not receive HB; * the retention time of each compound.

**Figure 6:**
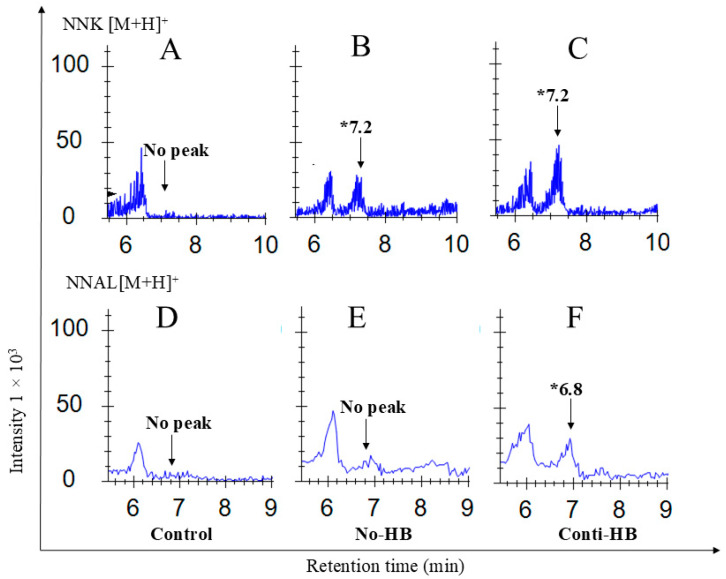
Detection of NNK and its major metabolites present in the serum of A/J mice. Blood samples were collected from the control, No-HB and Conti-HB groups following 24 h NNK challenge and analyzed by UPLC/ESI/Q-TOF/MS. Chromatograms are (**A**–**C**) NNK and (**D**–**F**) NNAL. HB, haskap berry; NNK, 4-(methylnitrosamino)-1-(3-pyridyl)-1-butanone; NNAL, 4-(methylnitrosamino)-1-(3-pyridyl)-1-butanol; * the retention time of each compound; Conti-HB mice received HB before and after NNK; No-HB mice did not receive HB, and the controls were administered an equal volume of saline i.p.

C3G, the major anthocyanin found in HB, its metabolites, NNK, and its primary metabolites were measured in the lung, liver, and serum of A/J mice after 24 h of NNK injection. C3G (Figure 5A–C) and cyanidin (Figure 5D–F), the aglycone of C3G, were detected in the lung and liver tissues of the HB-supplemented mice. Protocatechuic acid was detected in all the treatment groups, including the control group. NNK was present in both control diet-fed (No-HB) and HB supplement-fed mouse (Conti-HB) serum after 24 h of exposure (Figure 6A–C). However, the major secondary metabolite NNAL was detected only in HB-fed mouse tissues (Figure 6D–F).

### 2.5. The Binding Affinity of Structurally Diverse Ligands to CYP2A13, CYP2A6, and Cyp2a5

**Figure 7:**
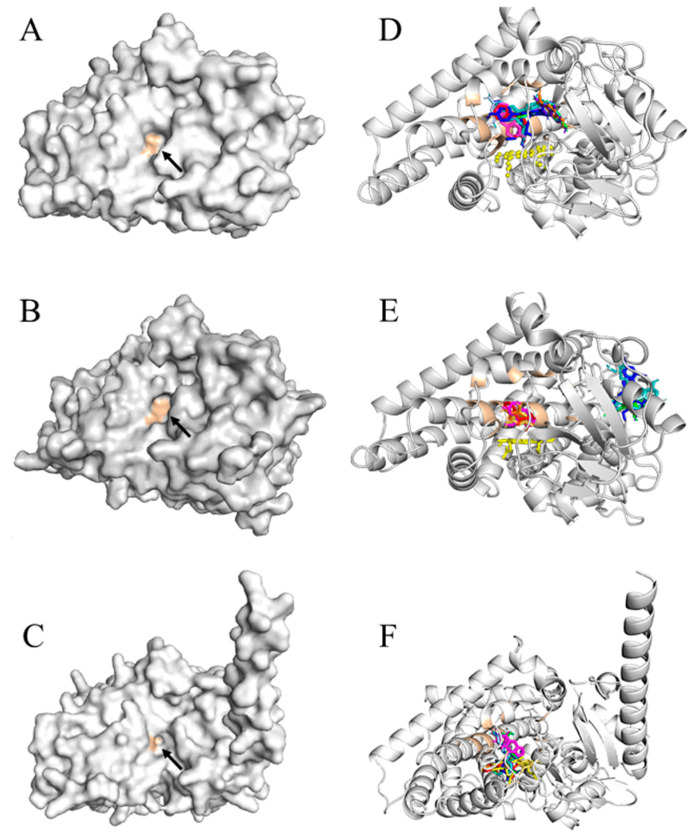
Ligand binding pocket of cytochrome P450 target proteins with bound ligand complexes. Panels to the left: (**A**) CYP2A13, (**B**) CYP2A6, and (**C**) Cyp2a5 enzymes are illustrated as surface colored by chain (in grey) with the active binding site (in tint color) indicated in black arrows. Panels to the right: (**D**) CYP2A13, (**E**) CYP2A6, and (**F**) Cyp2a5 proteins are represented in the ribbon model, and ligands are represented in the stick model. Cyanidin-3-*O*-glucoside, blue; cyanidin, purple, petunidin-3-*O*-glucoside, teal; petunidin, green; peonidin-3-*O*-glucoside, pale cyan; peonidin, cyan; phloroglucinaldehyde, yellow orange; protocatechuic acid, orange; 4-(methylnitrosamino)-1-(3-pyridyl)-1-butanone (NNK), red; 8-methoxypsoralen, magenta; HEME group, yellow. Figures (**A**–**F**) were generated using the PyMOL Molecular Graphics System, Version 2.0 Schrödinger, LLC, Mannheim, Germany.

Molecular docking was performed to predict the binding and inhibitory constants of HB flavonoids, NNK, control ligands, and inhibitors for mouse Cyp2a5 and human CYP2A13 and CYP2A6 (Table 1). The ligand-protein interactions and the distances between ligand-protein residues are presented in Supplementary Table S2. HB anthocyanins exhibited binding affinities between −5.5 and −10 kcal/mol, similar to the control substrate coumarin and inhibitor 8-MOP. C3G, petunidin-3-*O*-glucoside (Pt3G), and peonidin-3-*O*-glucoside (P3G) showed the highest affinity to Cyp2a5, followed by CYP2A13 and CYP2A6. Notably, Pt3G (−10.1 kcal/mol) had the strongest binding affinity to cyp2a5 compared to human CYP2A cytochromes. The aglycons, cyanidin, petunidin, and peonidin demonstrated the maximum binding affinity (−9.5 kcal/mol) and the lowest inhibition constant (0.11 µM) to CYP2A13 surpassing NNK. NNK-bound similarly to CYP2A13 and 2A6 (−7.7 kcal/mol). Anthocyanins had a greater affinity for CYP2A13 and Cyp2a5 than NNK (Table 1). The Ligand binding pocket of CYP proteins and predicted binding sites of anthocyanins, PCA, PGA, NNK, and 8-MOP are presented in Figure 7.

#### Ligand-Protein Interaction with P450 Enzymes

- CYP2A13

**Figure 8:**
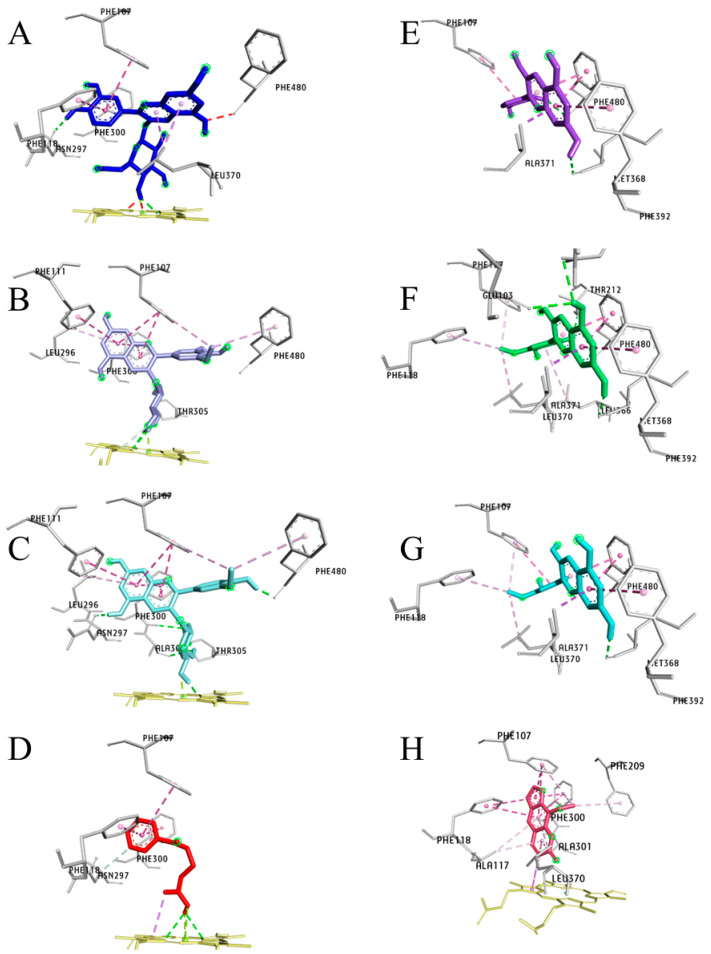
The highest-affinity C3G and its key metabolites are depicted along with NNK and 8-MOP, a known cytochrome 450 enzyme inhibitor in the three-dimensional plots and show the interaction with the active site of CYP2A13 enzyme. All the ligands (**A**) cyanidin-3-*O*-glucoside (C3G), (**B**) petunidin-3-*O*-glucoside (Pt3G), (**C**) peonidin-3-*O*-glucoside (P3G), (**D**) 4-(methylnitrosamino)-1-(3-pyridyl)-1-butanone (NNK), (**E**) cyanidin, (**F**) petunidin, (**G**) peonidin, and (**H**) 8-methoxypsoralen (8-MOP) are bound to the active site of CYP2A13 at their highest binding energy state. Color coding of ligand-amino acid interactions, green, conventional hydrogen bonds; pink, Pi bonds; purple, Sigma bonds; red, donor-donor bonds. Figure (**A**–**H**) are generated using BIOVIA Discovery Studio Visualizer V21.1.0.20198.

All three HB glycosides, C3G, Pt3G, and P3G (Figure 8A–C), displayed a similar orientation in the CYP2A13 binding pocket. Their glucose moiety was oriented perpendicular towards the heme group making hydrogen bonds, 2.05 Å, 2.60 Å, and 1.85 Å, respectively. The *N*-nitroso group of NNK was oriented towards the heme group and made two hydrogen bonds (3.06 Å and 3.13 Å) (Figure 8D). NNK, 8-MOP, and C3G shared almost identical amino acid residues, PhE107, Phe118, Asn297, Phe300, and heme moiety, except Pt3G had no interaction with Phe118 and Asn297 residues in the active site of CYP2A13. Asn297 forms a hydrogen bond with C3G (C4′-OH in B-ring, 2.04 Å), P3G (C5′-OH in A-ring 2.16 Å), and NNK (*N* of the pyrin ring 3.39 Å) as well as NAT and nicotine (Table 2). Cyanidin, petunidin, and peonidin demonstrated a comparable planner confirmation (Figure 8E–G) at their highest binding affinity and interacted with Phe107, Met368, Ala371, Phe392, and Phe480. Coumarin, the two phenolic metabolites of C3G, PCA, and PGA, and aglycones shared identical binding site residues (Ala371, Phe392, and Phe480).

- CYP2A6

**Figure 9:**
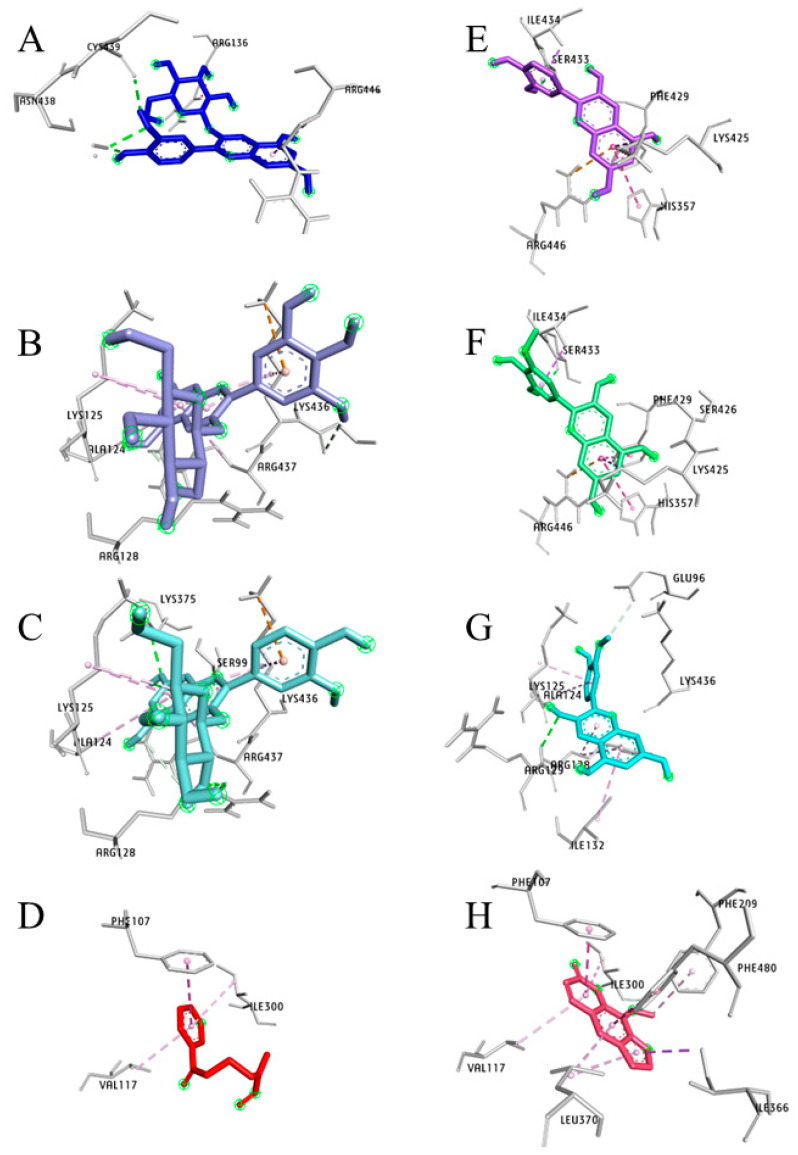
The high-affinity C3G and its metabolites are illustrated along with NNK and 8-MOP, a known cytochrome 450 enzyme inhibitor in the three-dimensional plots and show the interaction with the active site of the CYP2A6 enzyme. The ligands (**A**) cyanidin-3-*O*-glucoside (C3G), (**B**) petunidin-3-*O*-glucoside (Pt3G), (**C**) peonidin-3-*O*-glucoside (P3G), (**E**) cyanidin, (**F**) petunidin, and (**G**) peonidin are bound outside the active site of the CYP2A6 at their highest binding energy state. (**D**) 4-(methylnitrosamino)-1-(3-pyridyl)-1-butanone (NNK), and (**H**) 8-methoxypsoralen (8-MOP) are bound to the active site of CYP2A6. Color coding of ligand-amino acid interactions, green, conventional hydrogen bonds; pink, Pi bonds; purple, Sigma bonds; orange, Pi-Cation bond. Figures (**A**–**H**) are generated using BIOVIA Discovery Studio Visualizer V21.1.0.20198.

CYP2A6 has a confined hydrophobic active site with Asn297, which makes hydrogen bonds with ligands. The HB anthocyanins, C3G, Pt3G, P3G, cyanidin, petunidin, and peonidin were bound outside the active site of CYP2A6 at their highest binding affinity (Figure 7B,E and Figure 9). The CYP inhibitor, 8-MOP, intermingled with Phe107, Val117, Ile300, Ile366, and Phe480, whereas NNK shares Phe107, Val117, and Ile300 (Figure 9 and Table 2). Coumarin, the well-recognized CYP2A6 substrate and PCA and PGA interacted (Supplementary Figure S1) with active sites Val117, Asn297 and Leu370 and heme moiety of CYP2A6, forming hydrogen bonds with ASN297.

- Cyp2a5

**Figure 10:**
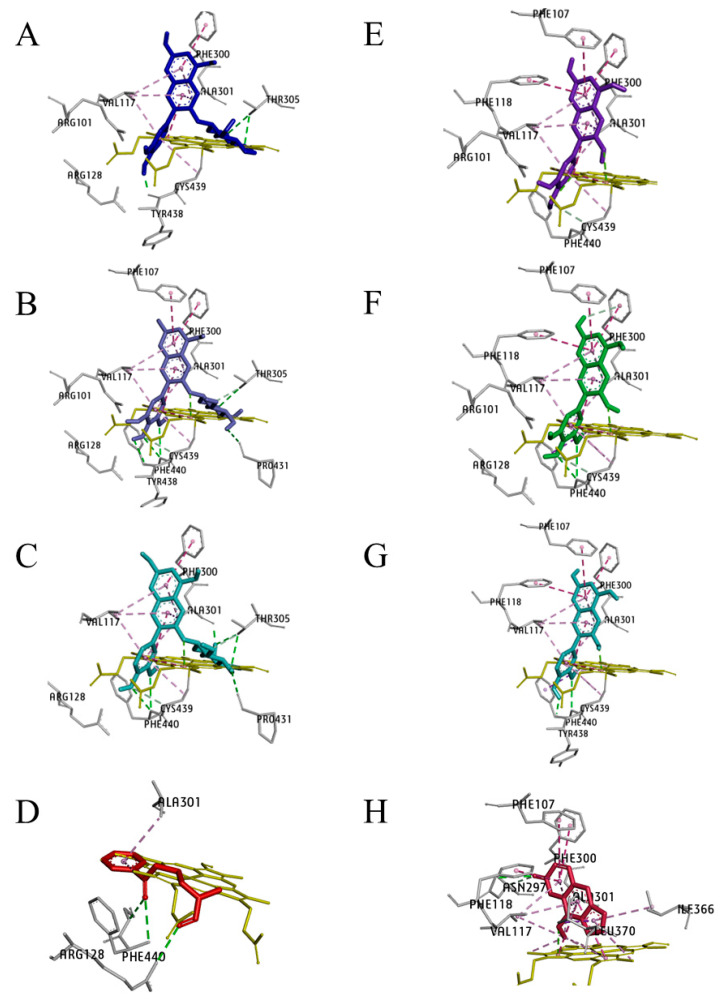
The high-affinity of C3G and its primary metabolites are shown along with NNK and 8-MOP, a known cytochrome 450 enzyme inhibitor in the three-dimensional plots and show the interaction with the active site of Cyp2a5 enzyme. All the ligands (**A**) cyanidin-3-*O*-glucoside (C3G), (**B**) petunidin-3-*O*-glucoside (Pt3G), (**C**) peonidin-3-*O*-glucoside (P3G), (**D**) 4-(methylnitrosamino)-1-(3-pyridyl)-1-butanone (NNK), (**E**) cyanidin, (**F**) petunidin, (**G**) peonidin and (**H**) 8-methoxypsoralen (8-MOP) are bound to the active site of CYP2A5 at their highest binding energy state. Colour coding of ligand-amino acid interactions, green, conventional hydrogen bonds; pink, Pi bonds; purple, Sigma bonds; red, donor-donor bonds. Figures (**A**–**H**) are generated using BIOVIA Discovery Studio Visualizer V21.1.0.20198.

The active site of Cyp2a5 has valine and ileum at residues 117 and 366 instead of alanine and leucine of CYP2A13. In contrast to CYP2A6, Cyp2a5 contains phenylalanine at residue 300 for ileum, alanine at residue 301 for glycine and methionine at 365 amino acids for valine. The enzyme-substrate binding and binding pocket of cyp2a5 is illustrated in Figure 7C, F. C3G, Pt3G, P3G, cyanidin, petunidin, and peonidin had a similar binding pattern with cyp2a5 along with 8-MOP and interacts with the heme group at C3’ (Table 2) at their highest affinity states. NNK interacted with Arg128, Ala301, Phe404, and heme cofactor of Cyp2a5 (Figure 10 and Table 2).

## 3. Discussion

Primarily CYP2A13 and CYP2A6 metabolize NNK in humans [15]. In mice, Cyp2a4 and Cyp2a5 generate NNK electrophilic metabolites in the lung and liver tissues [16]. Slowdown or inhibition of the carcinogen activation in lung and liver tissues could reduce the electrophilic metabolites and their subsequent genotoxicity and facilitate excretion without impairing human health. The present study evaluated whether HB dietary supplementation suppresses P450 expression, inhibits NNK electrophilic metabolite formation, and modulates NNK-activated inflammation. Interestingly, after the NNK injection, HB downregulated *cyp2a4* and *cyp2a5* mRNA expression 3- to 10-fold compared to the control and 3-fold compared to the NNK-injected regular diet-fed mice (No-HB). Flavonoid-rich plant extracts alter the expression of phase I enzymes; some upregulate their activities, while others downregulate or show no effect [17,18,19]. For instance, flavonoid galangin (8 mg/kg/d), upregulated *cyp1a2* and *cyp2b3* while downregulating *cyp2c13* and *cyp3a1* and has no effect on *cyp2c11*, *cyp2d4* and *cyp2e1* in Sprague Dawley rats after eight weeks [18]. In contrast, anthocyanins, including cyanidin (50 µM, 24 h) show no effect on P450 expression on human primary hepatocytes in vitro [20]. The mechanisms by which chemical agents and infections regulate Cyp2a5 are not entirely elucidated; particularly the specific function of each transcriptional regulator remains unclear [21].

PPARs are nuclear receptors and transcription factors that are involved in energy metabolism and directly induce certain P450 enzymes [22,23]; particularly, PPARα upregulates *cyp2a4* and *cyp2a5* in mice [24]. Previous studies have reported the direct ligand binding ability of C3G with PPARα to enhance its activity in C57BL/6J mice [22]. The C3G present in HB may have acted as a ligand for PPARα and induced the transcription of *cyp2a* in healthy mouse liver. In addition, PPARα has been suggested to be activated by AMPK to play a significant role in regulating hepatic lipid oxidation and thereby maintaining energy homeostasis [22,25] besides its role as a nuclear receptor [26,27,28]. The AMPK in HB-fed mice liver could have indirectly enhanced the PPARα activation as a transcription factor for *cyp2a5*. Therefore, HB could be modulating the expression of P450, particularly *cyp2a5* in mice by two pathways; first, C3G in HB could have acted as a direct PPARα ligand, and secondly, HB-induced AMPK may have transcriptionally induced the PPARα to activate *cyp2a* enzymes. The absence of data on Cyp2a4 and Cyp2a5 protein levels and activity are limitations of this study.

Many researchers have reported reduced expression and activity of P450s during infections and acute inflammations [29,30]. LPS (0.5 mg/kg body weight) downregulates the hepatic *cyp2a5* by 46% in DBA/2N mice [13]. Some evidence shows no direct effect of cytokines (IL-1β and IL-6) on *cyp2a5* mRNA and protein levels [13]. However, there is no proper evidence to develop a direct link between cytokines and cyp2a at the post-transcriptional level in the present study. Even though plant secondary metabolites are well reputed to prevent inflammation, in the current study, inflammatory mediators (TNF-α, not IL-6) were elevated in HB-fed healthy mice. We postulate from these data that the reduced expression of *cyp2a4* and *cyp2a5* may suppress the formation of the carcinogenic NNK metabolite while enhancing the formation of non-reactive, less potent metabolite NNAL via carbonyl reductases [1,31]. However, a quantitative analysis of these metabolites in the liver tissues would provide insight into the level of NNK metabolism in the liver of A/J mice with and without HB supplementation.

Besides the level of P450 enzyme expression in the tissues, ligand-enzyme interaction determines substrates’ orientation, binding affinity, and enzyme activity [32]. The plant compounds can act as substrates as well as inhibitors of P450 enzymes [33,34]. Therefore, along with the major HB anthocyanin C3G, its primary metabolites were further investigated by molecular docking to illustrate their binding affinities with CYP2A13, CYP2A6 and Cyp2a5. *N*′-nitrosoanatabine (NAT) and nicotine were selected since both are known tobacco smoke constituents, substrates, and competitive inhibitors of CYP2A enzymes. Coumarin is frequently considered a substrate for P450, whereas 8-methoxypsoralen (8-MOP) is an inhibitor [35,36].

The negative binding affinity indicates favorable spontaneous interactions between the ligand and the proteins [37]. NNK exhibited similar binding affinities to CYP2A13 and CYP2A6 (−7.7 kcal/mol) and slightly lower affinity to Cyp2a5 (−6.3 kcal/mol), consistent with previous reports from other scientists showing comparable binding affinities of NNK to CYP2A13 and CYP2A6 [15,38].

CYP2A13 has a conserved hydrophobic active site oriented on the heme group’s edge. Residue, Phe107, Phe111, Ala117, Phe118, Phe209, Leu296, Asn297, Phe300, Ala301, Glu304, Thr305, Met365, Leu366, Leu370, and Phe480 align the active site of CYP2A13 [32]. Asn297 is a key residue of CYP2A family enzymes as it offers hydrogen bonding with pyridine ring nitrogen and guides the orientation of the *N*-nitroso group towards the heme for methyl and methylene hydroxylation resulting in its activation [38,39]. Loss of this H-bond, due to Asn297 mutation, diminishes the metabolism of NNK, nicotine, and *N’*-nitrosonornicotine found in cigarette smoke [40]. The failure to produce hydrogen bonding between Asn297 of CYP2A6 and the pyridine ring could be a reason for the poor efficiency of NNK by CYP2A6 metabolism, as reported by many authors [15,32,41]. Based on the binding affinity, inhibition constant, and the NNK-P450 enzyme interactions, the efficiency of NNK activation could follow the order CYP2A13 > Cyp2a5 > CYP2A6. The orientation of the pyrin ring way from CYP2A13 heme active site may facilitate the methyl and methylene hydroxylation of NNK resulting in electrophilic metabolites generation in the human lung tissues.

Molecular docking suggests that flavonoids exhibit a stronger inhibitory effect on CYP2A13 than with CYP2A6 due to the hydrophobic bonds between the B-ring of flavonoids and CYP2A13 [8]. The key residues in CYP2A6 (Phe107, Phe111, Phe209, Leu366 and Leu370) and CYP2A13 (Leu296, Phe300, and Ala301) are crucial for inhibitor binding and strengthening non-polar interactions [42]. The substitution groups on C- and B-rings, particularly hydroxyl groups of flavonoids, reduce their inhibitory activity, as seen with myricetin against both CYP2A6 and CYP2A13. Additionally, a hydroxyl group at the C3 position of the C-ring also reduces the binding affinity and inhibition of quercetin, kaempferol and myricetin [33]. Docking results indicate methoxy groups on the B-ring and glucose at the C3 position influence metabolite orientation and ligand-protein interactions. A glucose moiety at C3 enhances Asn297-A-ring hydrogen bonding. Fewer hydroxyl groups and the methoxy group on the B-ring (P3G) also promote strong interactions with CYP2A13. Anthocyanidins showed the highest binding affinity with CYP2A13, likely due to the hydrogen at C4 of the C-ring. Among all metabolites, P3G > C3G > and aglycones may act as potential competitive inhibitors of CYP2A13, potentially suppressing NNK activation in smokers.

Cyp2a4 and Cyp2a5 metabolize the xenobiotics in mice with *cyp2a5* mRNA constituting about 90% of the total *cyp2a4* and *cyp2a5* mRNA [43]. NNK-derived electrophilic metabolites form methyl and methyl- and pyridylhydroxybutyl-DNA adducts in the liver of A/J mice [44]. Therefore, molecular docking was performed using Cyp2a5 protein structure to evaluate the anthocyanin-ligand interactions. The residues 209, 117, 305, 481 and 365 in Cyp2a4, and Cyp2a5 are necessary for direct ligand binding. Ala117 in Cyp2a4 regulates the catalytic activity of the enzyme [45]. Considering the binding affinities and ligand residue interactions Pt3G > C3G = P3G > petunidin and cyanidin could be potential cyp2a5 inhibitors which may suppress the NNK metabolism in mice liver. The substrate binding to P450 enzymes alters the electrostatic environment of the heme iron, causing shifts in the Soret band (approximately 417–450 nm). These changes reflect the transition of the heme iron’s electronic state and can be observed via absorption spectra [2,46]. Monitoring such spectral changes could strengthen the findings by providing direct evidence of substrate binding.

## 4. Materials and Methods

Well-ripened fresh HB (Brix value 14–16%) was collected from the Lone Tree Farm (New Canada, NS, Canada) and frozen at −20 °C. The lyophilized whole HB in powdered form was combined with regular mouse feed to prepare the HB dietary supplement and utilized in the mouse model.

### 4.1. Preparation of HB Dietary Supplement

Frozen HB was lyophilized under a 3600 mT vacuum at −20 °C for 72 h, ground to a fine powder, and stored at −80 °C. The HB dietary supplement was prepared according to our previous methods [9]. Ingesting 1.5 g of polyphenols per day is generally considered a health-promoting therapeutic dose for a healthy adult weighing around 70 kg [47,48]. A health-promoting animal equivalent dose, based on the human dose, was calculated using the following approach:

$$Human\;equivalent\;dose\;(mg/kg)\;=\;Animal\;dose(mg/kg)\times \frac{Animal\;Km\;factor}{Human\;Km\;factor}$$

The Km factor, calculated as body weight (kg) divided by body surface area (m^2^), is used to adjust doses across species. For mice, the Km factor is 3, while for adult humans, it is 37 [49].

Accordingly, the experimental diet for each mouse per day consisted of 0.2 g of HB powder, providing an equivalent of 6 mg of C3G per mouse per day, mixed into standard mouse chow (Prolab^®^ RMH 3000 from LabDiet, Richmond, IN, USA) and formed into pellets (2 g dry weight/pellet). Pellets were prepared every two days and stored in sealed containers in the dark at 4 °C. The control diet consisted of standard mouse chow.

### 4.2. In Vivo Mouse Model

Female A/J albino mice (4–6 weeks) were purchased from the Jackson Laboratory, Bar Harbor, ME, USA. In vivo, the study was performed at the Life Science Research Institute, Summer Street, Halifax, NS, Canada, following the approval of the Dalhousie University Committee on Laboratory Animals (protocol: 2020-09).

#### 4.2.1. Determine the Biological Activity of the HB Diet Against NNK-Induced Lung Tumorigenesis

**Figure 11:**
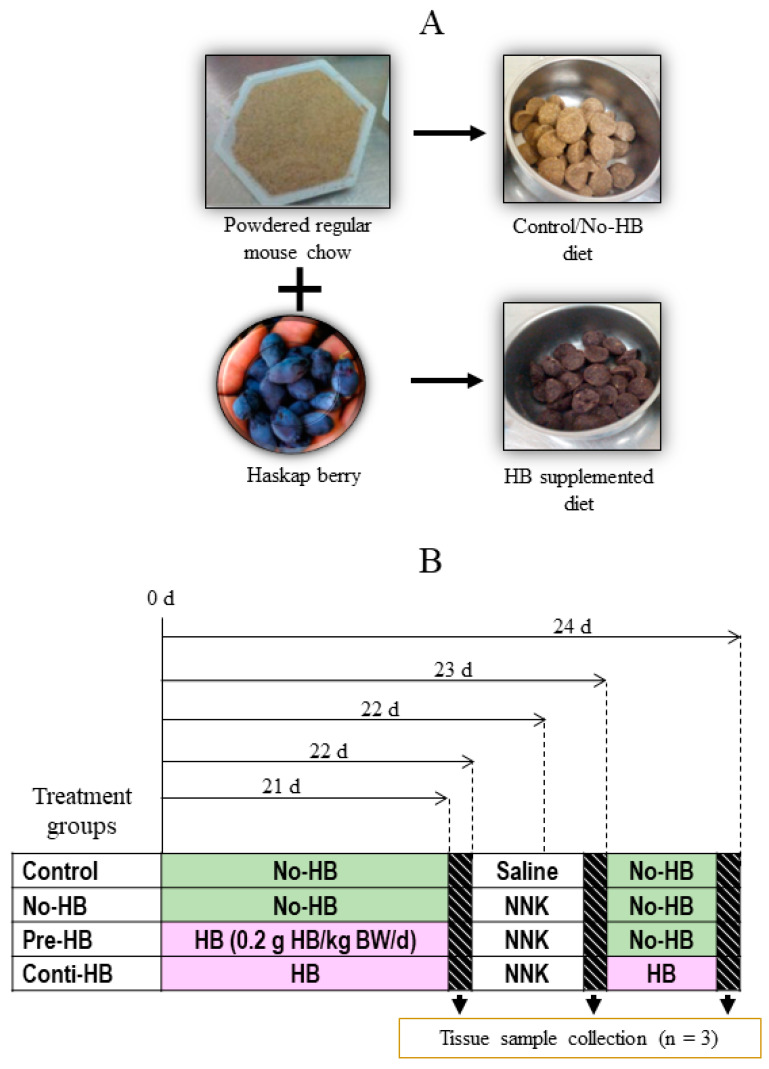
Experimental procedure of A/J mouse model. (**A**) Haskap berry (HB)-rich diet (6 mg of C3G in 0.2 g of HB/mouse/day) was prepared by mixing powdered regular mouse chow with freeze-dried HB powder and formed into pellets. A control/no-HB diet was made using regular mouse chow without mixing HB. (**B**) Experimental groups and timeline. Control/No-HB diet was given to the control and No-HB groups throughout the experimental period. The Pre-HB group received an HB-rich diet (6 mg of C3G in 0.2 g of HB/mouse/day) only before the NNK injection, while the Conti-HB group received HB diet even after the NNK injection. A single intraperitoneal injection of NNK (100 mg/kg BW) was used to induce lung carcinogenesis in the no-HB, pre-HB and Conti-HB groups, and saline was used as a shame for the control group. Three mice from each group were euthanized after 21 days before the NNK injection, 24 h and 72 h after the NNK injection. Blood and liver tissues were collected for analysis.

A total of 36 mice (n = 9) were housed individually in filter-topped plastic cages and maintained under a 12 h light-dark cycle. Prolab^®^ RMH 3000 diet and distilled water were provided ad libitum. After one week of adaptation, mice were randomly divided into four experimental groups (n = 9) and tissues were collected from three mice (n = 3) at each time point. The HB-incorporated experimental or control diet was given daily as a dietary supplement, as presented in Figure 11. The control diet was given to the mice in control and No-HB groups, while Pre-HB and Conti-HB groups were fed with HB incorporated experimental diet for 21 days. NNK (100 mg/kg body weight as a single intraperitoneal injection) was injected into the mice in No-HB, Pre-HB and Conti-HB groups on the following day (day 22) to induce lung tumorigenesis, but the same volume of saline was injected into the control mice. Following the NNK challenge (day 23 onwards), the control diet was given to the control, No-HB and Pre-HB groups, while the experimental diet was fed to the Conti-HB group for another 3 days (Figure 11B). The body weight of each mouse was measured weekly, and their behavioral and performance changes were monitored daily. Three mice from each group were euthanized just before NNK injection and 24 h and 72 h after NNK injection. Tissue samples were collected, cryopreserved, and stored at −80 °C until further analysis.

#### 4.2.2. Western Blotting

Liver tissues were ground in liquid nitrogen and then lysed in ice-cold RIPA lysis buffer containing freshly added protease inhibitors (1 mM phenylmethylsulfonylfluoride, 10 μg/ mL aprotinin, 5 μg/mL leupeptin, 10 μM phenylarsine oxide, 1 mM dithiothreitol and 5 μg/mL pepstatin) for 15 min to collect cytoplasmic proteins. The tissue lysates were collected at 14,000× *g* for 10 min, and the protein concentration was determined by the Bradford assay (Bio-Rad Laboratories, Inc., Mississauga, ON, Canada). Proteins were denatured using the Blue Loading Buffer (New England BioLabs™, Ipswich, MA, USA) and standardized with the sample buffer. Equal protein amounts (10–20 μg) were loaded onto 10% polyacrylamide gel cassettes and subjected to electrophoresis for 40 min at 200 V. The proteins were then transferred to nitrocellulose membranes, and the blots were blocked for 1 h in 5% skim milk dissolved in Tween-TBS buffer (0.25 M Tris [pH 7.5], 150 mM NaCl, and 0.2% Tween-20) to prevent non-specific binding. The antibodies phospho-AMPKα (Thr172) (cat. no. 8208S) from New England BioLabs Ltd. Whitby, ON, Canada was used for protein analysis. The blots were then thoroughly washed with Tween-TBS and incubated with an HRP-conjugated donkey anti-rabbit or anti-mouse IgG secondary antibody for 1 h. Uniform protein loading was verified by probing the blots with an HRP-conjugated rabbit anti-actin antibody. Target proteins were visualized using enhanced chemiluminescence (ECL) with the Clarity™ and Clarity Max™ Western ECL Substrates Kit (Bio-Rad Laboratories Inc., Hercules, CA, USA).

#### 4.2.3. Quantitative Reverse Transcription PCR (RT-qPCR)

The liver samples were homogenized in liquid nitrogen and stored at −80 °C until mRNA isolation. According to the manufacturer’s instructions, about 40 mg of sample from each liver was weighed for RNA isolation using the Aurum total RNA isolation kit (cat. no. 7326820, BioRad Laboratories Inc., Mississauga, ON, Canada). The amount of total RNA was quantified by spectrophotometric OD_260/280_ measurement. The cDNA was synthesized using iScript cDNA Synthesis Kit (BioRad Laboratories Inc., Mississauga, ON, Canada) and stored at −20 °C until analysis. For cDNA synthesis, 1 µg of total RNA was incubated with the 5× iScript reaction mixture (4 µL), reverse transcriptase (1 µL), and nuclease-free water (5 µL) according to the manufacturer’s guidelines in a total volume of 20 µL. The mixture was vortexed briefly and incubated at 25 °C for 5 min for priming, then at 46 °C for 20 min for reverse transcription and at 95 °C for 1 min for the inactivation of enzymes in CFX Connect^TM^ Real-Time PCR detection system (BioRad Laboratories Inc., Mississauga, ON, Canada).

The primer sequences and probes are shown in Supplementary Table S2. The PCR reactions were prepared in a final volume of 20 µL, with a final concentration of 2× SsoAdvanced^TM^ Universal SYBR^®^ Green Supermix (BioRad Laboratories Inc., Mississauga, ON, Canada) with 500 nm each forward and reverse primer and nuclease-free water. Then, 50 ng of cDNA was mixed, and processed under the following conditions. The thermal cycling condition comprised an initial incubation for polymerase activation and cDNA denaturation at 95 °C for 30 s, followed by 40 annealing cycles and extension at 60 °C for 30 s. Each measurement was recorded in triplicate, and the threshold cycle (Ct) was documented. A no-template control was included, along with a standard curve generated from four to five serial dilutions of cDNA. mixture was performed for all runs. Two endogenous control genes, actin and GADPH, were used in each plate. Data were analyzed by XP514-CM22-CFXMaestro2.2 software from BioRad Laboratories Inc., Mississauga, ON, Canada.

#### 4.2.4. Ultra Performance Liquid Chromatography/Electrospray Ionization-Quadrupole-Time of Flight-Mass Spectrometry (UPLC/ESI/MS-Q-TOF) Analysis

The metabolites of NNK and HB anthocyanins in lung tissues were analyzed by UPLC/ESI/MS-Q-TOF method at the Biological Mass Spectrometry Core Facility, Life Science Research Institute, Summer Streat, Halifax, NS, Canada. Approximately 20–30 mg of lung tissue from each mouse was weighed and homogenized in PBS (1:5, *w*/*v*) on ice. The homogenate was then centrifuged at 14,000× *g* for 15 min at 4 °C, and the supernatant was collected. A 500 μL aliquot of the homogenate was mixed with two parts of cold acetonitrile and acetone (80:20, *v*/*v*) and stored at 4 °C overnight to aid protein precipitation. The mixture was centrifuged again at 14,000× *g* for 15 min at 4 °C; the supernatant was concentrated under a gentle stream of nitrogen gas. The resultant was reconstituted in methanol, and the UPLC/ESI/MS-Q-TOF was used for metabolite analysis [50,51].

### 4.3. Molecular Docking

The human CYP2A13 protein structure complexed with two molecules of NNK (PDB 4EJI) with a resolution of 2.10 Å and CYP2A6 complexed with nicotine (PDB 4EJJ) with a resolution of 2.30 Å [36] were obtained from the protein data bank. The mouse cyp2a5 protein (P20852) amino acid sequence [52,53] was obtained from the AlphaFold2 UniProt databank. Then a heme-bound cyp2a5 AlphaFold3 protein structure was predicted in Google DeepMind’s newly launched AlphaFold webserver and confidence metrics were determined [54]. The predicted local distance difference test (pLDDT) score was above 90 for the predicted protein. Further, the predicted template modelling (pTM) and the interface predicted template modelling (ipTM) scores were 0.91 and 0.95, respectively. The newly predicted cyp2a5 model was further validated with its homologous CYP2A13 in SWISS-MODEL server (Swiss Institute of Bioinformatics, Basel, Switzerland) and presented in Supplementary Table S3 and Figure S2. The unwanted ligands and water molecules were deleted, polar hydrogen was added, and non-polar hydrogen disappeared from the crystal structure of P450 using the Discovery Studio Visualizer v21.1.0.20298 (BIOVIA, San Diego, CA, USA) program. The structures of flavonoids, NNK, nicotine, *N*′-nitrosoanatabine (NAT), coumarin and 8-MOP were obtained from the PubChem database (Figure 1). Ligand-free crystal structure of protein and each ligand were analyzed to determine the protein-ligand interactions using the PyRx-Virtual Screening Tool software coupled with AutoDock Vina version 0.8 (PyRx-Python Prescription, The Scripps Research Institute, San Diego, CA, USA). The grid boxes with 25 × 25 × 25 point for CYP2A13, 40 × 40 × 40 for cyp2a5 and 36 × 36 × 36 for CYP2A6 that fully encompassed the active site was employed to determine the ligand-receptor interactions. The inhibition constant (Ki) was calculated from the binding affinity (ΔG) using the equation: K_i_ = exp (ΔG/RT) where R is the universal gas constant (1.985 × 10^−3^ kcal/mol/K) and T is the temperature (298.15 K) [14]. In future, some of the limitations of the present investigation such as optical absorption spectral changes of purified P450 caused by substrate binding need to be determined.

## 5. Conclusions

In conclusion, this study demonstrated that haskap berry (HB) supplementation upregulates the expression of *cyp2a4* and *cyp2a5* mRNA in hepatic tissues when deprived of the procarcinogen NNK. This transcriptional activation of P450 may be mediated by the activation of PPARα, potentially driven by C3G, the primary anthocyanin compound in HB. Conversely, in the presence of NNK, HB supplementation downregulated *cyp2a4* and *cyp2a5* expression and facilitated the formation of non-carcinogenic NNK metabolites. There is insufficient data to determine the immunomodulatory effects of HB on liver tissue in NNK-exposed mice. Molecular docking analyses highlighted strong hydrophobic interactions between C3G, its major metabolite P3G, Pt3G, and cyanidin with CYP2A13, followed by *cyp2a5*. These findings suggest that HB anthocyanins, as dietary compounds, may inhibit NNK activation by CYP2A13 in the respiratory tract, potentially reducing lung carcinogenesis in smokers. However, further clinical studies are necessary before recommending these natural compounds as therapeutic agents for managing lung cancer.

## Figures and Tables

**Table 1:** The binding affinity and inhibition constant of cyanidin-3-*O*-glucoside and its metabolites, carcinogenic and non-carcinogenic xenobiotics, with CYP2A13, CYP2A6 and Cyp2a5 enzymes.

Ligand Molecule	Binding Affinity (kcal/mol)			Inhibition Constant (Ki) (µM)		
	CYP2A13	CYP2A6	Cyp2a5	CYP2A13	CYP2A6	Cyp2a5
C3G and its metabolites						
C3G	−8.6	−6.8	−9.8	0.49	10.23	0.06
Cyanidin	−9.5	−6.5	−8.9	0.11	16.99	0.29
Pt3G	−8.3	−6.5	−10.1	0.81	16.99	0.04
Petunidin	−9.5	−6.5	−9.2	0.11	16.99	0.18
P3G	−8.3	−6.6	−9.8	0.81	14.35	0.06
Peonidin	−9.5	−6.4	−8.8	0.11	20.12	0.35
PGA	−6.6	−6.3	−5.5	14.35	23.82	92.0
PCA	−6.8	−6.9	−5.9	10.23	8.64	46.8
NNK and cigarette smoke constituents						
NNK	−7.7	−7.7	−6.3	2.24	2.24	23.8
NAT	−7.5	−8	−7.1	3.14	1.35	6.16
Nicotine	−7	−7.5	−7.1	7.30	3.14	6.16
A common ligand and an inhibitor of P450						
Coumarin ^a^	−8.5	−7.9	−7.3	0.58	1.60	4.4
8-MOP ^a^^a^	−8.5	−7	−7.5	0.58	7.3	3.14

The binding affinity was determined by molecular docking using PyRx-Python Prescription 0.8 [2008–2010]. The inhibition constant (Ki) was calculated from the binding affinity (ΔG) using the formula: Ki = exp (ΔG/RT), where R is the universal gas constant (1.985 × 10^−3^ kcal/mol/K), and T is the temperature (298.15 K) [14]. C3G, cyanidin-3-*O*-glucoside; Pt3G, petunidin-3-*O*-glucoside; P3G, peonidin-3-*O*-glucoside; PGA, phloroglucinaldehyde; PCA, protocatechuic acid; NNK, 4-(methylnitrosamino)-1-(3-pyridyl)-1-butanone; NAT, *N*′-nitrosoanatabine; 8-MOP, 8-methoxypsoralen, ^a^ known P450 ligand and ^a^^a^ known P450 inhibitor.

**Table 2:** The key interacting amino acids of CYP2A13, CYP2A6 and Cyp2a5 enzymes with cyanidin-3-*O*-glucoside and its major metabolites, carcinogenic, and non-carcinogenic xenobiotics.

Molecule Name	CYP2A13	CYP2A6	Cyp2a5
C3G and its metabolites			
C3G	Phe107, Phe118, Asn297, Phe300, Leu370, Heme	Asn438, Cys439, Arg446	Val116, Phe300, Ala301, Thr305, Cys439, Heme
Cyanidin	Phe107, Met368, Ala371, Phe392, Phe480	His357, Lys425, Phe429, Ser433, Ile434, Asn438, Arg446	Phe107, Val117, Phe118, Phe300, Ala301, Cyc439, Heme
Pt3G	Gln104, Phe107, Phe111, Leu296, Phe300, Phe480, Heme	Ala124, Lys125, Arg128, Gly435, Lys436, Arg437	Phe107, Cal117, Phe300, Ala301, Cys439, Heme
Petunidin	Glu103, Phe107, Phe118, Thr212, Glu221, Leu366, Met368, Leu370, Ala371, Phe392, Phe480	Val92, His357, Lys425, Phe429, Ser433, Ile434, Asn438, Arg446, Ser426	Phe107, Val117, Phe118, Phe300, Ala301, Cys439, Phe440. Heme
P3G	Gln104, Phe107, Phe111, Leu296, Asn297, Phe300, Ala301, Thr305, Phe480, Heme	Ser99, Ala124, Lys125, Arg128, Lys375, Lys436, Arg437	Val117, Phe300, Ala301, Thr305, Pro431, Cys439, Phe440, Heme
Peonidin	Phe107, Phe118, Met368, Leu370, Ala371, Phe392, Phe480	Glu96, Ala124, Lys125, Arg128, Arg129, Ile132, Lys436	Phe107, Val117, Phe118, Phe300, Ala301, Tyr438, Cys439, Phe440. Heme
PGA	Glu103, Met368, Gly369, Ala371, Phe392, Phe480	Val117, Asn297, Leu370, Heme	Arg101, Leu370, Ser433, Pro431
PCA	Gyl369, Ala371, Phe392, Phe480	Val117, Asn297, Leu370	Arg101, Leu370, Arg372, Tyr438, Cys439
NNK and cigarette smoke constituents			
NNK	Phe107, Phe118, Asn297, Phe300, Heme, Fe	Phe107, Val117, Ile300	Arg128, Ala301, Phe440, Gly441, Heme
NAT	Phe107, Phe118, Asn297, Phe300, Ala301, Leu366, Leu370, Heme, Fe	Phe107, Val117, Phe209, Ile300, Gly301, Thr305, Ile366	Phe107, Val117, Phe118, Asn297, Phe300, Ala301, Ile366, Leu370, Phe480, Heme
Nicotine	Phe107, Phe118, Asn297, Phe300, Leu370	Phe107, Val117, Ile300	Phe107, Val117, Phe118, Phe209, Phe300, Ala301, Phe480
Common ligand and inhibitor of P450			
Coumarin ^a^	Ala371, Phe392, Phe480	Val117, Asn297, Leu370	Phe107, Val117, Phe118, Phe300, Ala301
8-MOP ^a^^a^	Phe107, Ala117, Phe118, Phe209, Phe300, Ala301, Leu370, Heme	Phe107, Val117, Phe209, Ile300, Ile366, Leu370, Phe480	Phe107, Val117, Phe118, Asn297, Phe300, Ala301, Ile366, Leu370, Heme

C3G, cyanidin-3-*O*-glucoside; Pt3G, petunidin-3-*O*-glucoside; P3G, peonidin-3-*O*-glucoside; PGA, phloroglucinaldehyde; PCA, protocatechuic acid; NNK, 4-(methylnitrosamino)-1-(3-pyridyl)-1-butanone; NAT, *N*′-nitrosoanatabine; 8-MOP, 8-methoxypsoralen; ^a^ Known P450 ligand and ^a^^a^ known P450 inhibitor.

## Data Availability

All the data are presented in the paper.

## Supplementary Materials

The following supporting information can be downloaded at: https://www.mdpi.com/article/10.3390/ph17121615/s1, Table S1: The list of forward and reverse primers used to analyze the gene expression by RT-PCR; Table S2: The type of ligand-protein interactions and atoms involved in the active site of P450 proteins; Table S3: The results of the structure validation in SWISS-Model server for predicted AlphaFold 3 cyp2a5 in reference to CYP2A13; Figure S1: The high-affinity PCA and PGA, two primary phenolic metabolites of C3G, are shown in the three-dimensional plots; Figure S2: The quality assessment of heme-bound cyp2a5 AlphaFold 3 prediction with its homology CYP2A13 in SWISS-MODEL structure assessment and modelling server.

## Abbreviations

P450; cytochrome P450; HB; haskap berry; AMPK; AMP-activated protein kinase; PPARα; peroxisome proliferator-activated receptor-alpha; PCA; protocatechuic acid; PGA; phlorogucinaldehyde; C3G; cyanidin-3-*O*-glucoside; NNK; 4-(methylnitrosamino)-1-(3-pyridyl)-1-butanone; Pt3G; petunidin-3-*O*-glucoside; P3G; peonidin-3-*O*-glucoside; NAT; *N’*-nitrosoanatabine; 8-MOP; 8-methoxypsoralen.
