# Supplementary material for: Suppression of NNK Metabolism by Anthocyanin-Rich Haskap Berry Supplementation Through Modulation of P450 Enzymes
Source: Pharmaceuticals (Basel). 2024 Nov 30;17(12):1615. doi: 10.3390/ph17121615 (PMC11728747; doi:10.3390/ph17121615)
Supplement: Supplementary file 1 [file pharmaceuticals-17-01615-s001.zip › pharmaceuticals-3291917-supplementary.pdf]

SUPPLEMENTARY DOCUMENTS

Supplementary Table S1. The list of forward and reverse primers used to analyze the gene expression by RT-PCR

**Marker**	**Accession no**	**Forward 5' – 3'**	**Reverse 5' -3'**
CYP2A4	NM_009997.2	GGAAGACGAACGGTGCT TTC	TTCCCAGCATCA TTCTAAGA
CYP2A5	NM_007812.4	GGAAGACGAACGGTGCT TTT	TTCCCAGCATCA TTCGAAGC
IL-6	NM_031168	CTGCAAGAGACTTCCATC CAG	AGTGGTATAGAC AGGTCTGTTGG
TNF-α	NM_013693	CAGGCGGTGCCTATGTCT C	CGATCACCCCGA AGTTCAGTAG
GAPDH	NM_008084.3	GGGAAGCCCATCACCATC TT	GCCTTCTCCATG GTGGTGAA
Actin	NM_007393.5	CTCTGGCTCCTAGCACCA TGAAGA	GTAAAACGCAG CTCAGTAACAGT CCG

Supplementary Table S2. The type of ligand-protein interactions and atoms involved in the active site of P450 proteins.

CYP2A13				
**Ligand**	**Type of ligand-protein interaction**	**Distance (Å)**	**Donar molecule**	**Recipient molecule**
C3G	Hydrogen Bond	2.05915	C3G	HEM
	Hydrogen Bond	2.42102	C3G	ASN297
	Pi-Sigma	3.61782	LEU370	C3G
	Pi-Sigma	3.45054	LEU370	C3G
	Pi-Lone Pair	1.99524	C3G	HEM
	Pi-Pi Stacked	4.47568	PHE300	C3G
	Pi-Pi T-shaped	4.69452	PHE107	C3G
	Pi-Pi T-shaped	5.27342	PHE118	C3G
Cyanidin	Hydrogen Bond	2.58724	C3G	PHE480
	Hydrogen Bond	2.49553	C3G	MET368
	Pi-Sigma	3.91794	ALA371	Cyanidin
	Pi-Pi Stacked	3.90114	PHE480	Cyanidin
	Pi-Pi Stacked	3.96933	PHE480	Cyanidin
	Pi-Pi T-shaped	4.80974	PHE107	Cyanidin
	Pi-Pi T-shaped	4.8865	PHE392	Cyanidin
Pt3G	Hydrogen Bond	2.60476	HEM501	Pet3G
	Hydrogen Bond	2.14202	Pet3G	HEM501
	Carbon Hydrogen Bond	3.44712	Pet3G	GLN104
	Pi-Donor Hydrogen Bond	3.07664	Pet3G	HEM
	Pi-Lone Pair	1.94857	Pet3G	HEM
	Pi-Pi Stacked	4.12007	PHE300	Pet3G
	Pi-Pi Stacked	3.7585	PHE300	Pet3G
	Pi-Pi T-shaped	4.43066	PHE107	Pet3G
	Pi-Pi T-shaped	5.02827	PHE107	Pet3G
	Pi-Pi T-shaped	5.02039	PHE111	Pet3G
	Pi-Alkyl	5.41656	PHE107	Pet3G
	Pi-Alkyl	4.85304	PHE480	Pet3G
	Pi-Alkyl	5.22302	Pet3G	LEU296
Petunidin	Hydrogen Bond	2.14546	Petunidin	THR212
	Hydrogen Bond	2.91069	Petunidin	GLU103
	Hydrogen Bond	2.77161	Petunidin	GLU221
	Hydrogen Bond	2.1847	Petunidin	MET368
	Pi-Sigma	3.95863	ALA371	Petunidin
	Pi-Pi Stacked	3.90542	PHE480	Petunidin
	Pi-Pi Stacked	3.90585	PHE480	Petunidin

	Pi-Pi T-shaped	4.89379	PHE392	Petunidin
	Alkyl	4.69935	Petunidin	LEU370
	Pi-Alkyl	4.1773	PHE107	Petunidin
	Pi-Alkyl	4.72284	PHE118	Petunidin
	Pi-Alkyl	5.48873	Petunidin	LEU366
P3G	Hydrogen Bond	1.96385	THR305	Peo3G
	Hydrogen Bond	2.70737	Peo3G	PHE300
	Hydrogen Bond	2.95406	Peo3G	ALA301
	Hydrogen Bond	1.84532	Peo3G	HEM
	Hydrogen Bond	1.69427	Peo3G	PHE480
	Hydrogen Bond	2.16585	Peo3G	ASN297
	Carbon Hydrogen Bond	3.51138	Peo3G	GLN104
	Pi-Lone Pair	1.92848	Peo3G	HEM
	Pi-Pi Stacked	4.09426	PHE300	Peo3G
	Pi-Pi Stacked	3.74826	PHE300	Peo3G
	Pi-Pi T-shaped	4.4341	PHE107	Peo3G
	Pi-Pi T-shaped	5.00897	PHE107	Peo3G
	Pi-Pi T-shaped	5.02669	PHE111	Peo3G
	Pi-Alkyl	5.22543	PHE107	Peo3G
	Pi-Alkyl	4.91246	PHE480	Peo3G
	Pi-Alkyl	5.23432	Peo3G	LEU296
Peonidin	Hydrogen Bond	2.14204	Peonidin	MET368
	Pi-Sigma	3.96881	ALA371	Peonidin
	Pi-Pi Stacked	3.88412	PHE480	Peonidin
	Pi-Pi Stacked	3.89221	PHE480	Peonidin
	Pi-Pi T-shaped	4.7649	PHE107	Peonidin
	Pi-Pi T-shaped	4.9137	PHE392	Peonidin
	Alkyl	4.74278	Peonidin	LEU370
	Pi-Alkyl	4.24051	PHE107	Peonidin
	Pi-Alkyl	4.82769	PHE118	Peonidin
PGA	Hydrogen Bond	2.6374	PGA	MET368
	Hydrogen Bond	2.24201	PGA	GLU103
	Carbon Hydrogen Bond	3.18887	PGA	GLY369
	Pi-Pi Stacked	3.74987	PHE480	PGA
	Pi-Pi T-shaped	5.75343	PHE392	PGA
	Pi-Alkyl	4.34171	PGA	ALA371
PCA	Hydrogen Bond	1.97376	PCA	GLY369
	Pi-Pi Stacked	3.64036	PHE480	PCA
	Pi-Pi T-shaped	5.44723	PHE392	PCA

	Pi-Alkyl	4.26989	PCA	ALA371
NNK	Hydrogen Bond	3.13819	HEM501	NNK
	Hydrogen Bond	3.06613	HEM	NNK
	Carbon Hydrogen Bond	3.39841	ASN297	NNK
	Metal-Acceptor	2.22409	HEM	NNK
	Pi-Sigma	3.83745	NNK	HEM
	Pi-Lone Pair	2.99067	NNK	HEM
	Pi-Lone Pair	2.25451	NNK	HEM
	Pi-Pi Stacked	4.12328	PHE300	NNK
	Pi-Pi T-shaped	4.99841	PHE107	NNK
	Pi-Pi T-shaped	5.65541	PHE118	NNK
NAT	Hydrogen Bond	3.05195	ALA301	NAT
	Carbon Hydrogen Bond	3.71039	NAT	ASN297
	Carbon Hydrogen Bond	3.53742	NAT	HEM
	Pi-Sigma	3.98552	ALA301	NAT
	Pi-Pi T-shaped	5.1286	HEM	NAT
	Pi-Pi T-shaped	4.71948	NAT	HEM
	Pi-Alkyl	4.54425	PHE107	NAT
	Pi-Alkyl	5.33409	PHE118	NAT
	Pi-Alkyl	4.54087	PHE300	NAT
	Pi-Alkyl	5.44449	NAT	LEU366
	Pi-Alkyl	5.34141	NAT	LEU370
Nicotine	Carbon Hydrogen Bond	3.46846	ASN297	Nicotine
	Pi-Pi Stacked	4.05042	PHE300	Nicotine
	Pi-Pi T-shaped	4.98354	PHE107	Nicotine
	Pi-Pi T-shaped	5.72473	PHE118	Nicotine
	Alkyl	5.03456	LEU370	Nicotine
	Pi-Alkyl	4.87144	PHE107	Nicotine
Coumarin	Pi-Pi Stacked	3.96763	PHE480	Coumarin
	Pi-Pi Stacked	3.68455	PHE480	Coumarin
	Pi-Pi T-shaped	5.20359	PHE392	Coumarin
	Pi-Alkyl	4.13725	Coumarin	ALA371
8-MOP	Pi-Sigma	3.53312	8-MOP	HEM
	Pi-Pi Stacked	4.67938	PHE300	8-MOP
	Pi-Pi Stacked	3.76189	8-MOP	PHE300
	Pi-Pi T-shaped	5.48805	PHE107	8-MOP
	Pi-Pi T-shaped	5.7055	PHE118	8-MOP
	Pi-Pi T-shaped	4.9165	HEM	8-MOP

	Pi-Pi T-shaped	4.45016	8-MOP	PHE107
	Pi-Pi T-shaped	5.88585	8-MOP	PHE118
	Pi-Alkyl	4.81344	PHE107	8-MOP
	Pi-Alkyl	5.04123	PHE209	8-MOP
	Pi-Alkyl	4.52002	PHE300	8-MOP
	Pi-Alkyl	4.50848	8-MOP	ALA117
	Pi-Alkyl	4.03474	8-MOP	ALA301
	Pi-Alkyl	5.08812	8-MOP	LEU370
	Pi-Alkyl	5.32263	8-MOP	ALA117
	Pi-Alkyl	5.01313	8-MOP	ALA301
		CYP2A6		
C3G	Hydrogen Bond	2.53422	ASN438	C3G
	Hydrogen Bond	1.87581	ASN438	C3G
	Hydrogen Bond	2.28918	C3G	CYS439
	Pi-Alkyl	5.24232	C3G	ARG446
Cyanidin	Hydrogen Bond	2.07823	ASN438	Cyanidin
	Hydrogen Bond	2.26733	Cyanidin	SER433
	Pi-Cation; Pi-Donor Hydrogen Bond	4.12412	ARG446	Cyanidin
	Pi-Sigma	3.3924	LYS425	Cyanidin
	Pi-Sigma	3.9498	ILE434	Cyanidin
	Pi-Pi Stacked	5.28018	HIS357	Cyanidin
	Pi-Pi T-shaped	5.45548	PHE429	Cyanidin
Pt3G	Carbon Hydrogen Bond	3.77458	ARG128	Pt3G
	Carbon Hydrogen Bond	3.58106	Pt3G	GLY435
	Pi-Cation	4.73599	LYS436	Pt3G
	Alkyl	4.73324	Pt3G	LYS436
	Pi-Alkyl	5.49304	Pt3G	LYS125
	Pi-Alkyl	4.39098	Pt3G	LYS436
	Pi-Alkyl	5.11103	Pt3G	ALA124
	Pi-Alkyl	4.74111	Pt3G	LYS125
	Pi-Alkyl	5.14007	Pt3G	LYS436
	Pi-Alkyl	4.9601	Pt3G	ARG437
	Pi-Alkyl	4.0731	Pt3G	LYS436
Petunidin	Hydrogen Bond	1.9365	ASN438	Petunidin
	Hydrogen Bond	2.56492	Petunidin	SER433
	Pi-Cation; Pi-Donor Hydrogen Bond	4.05996	ARG446	Petunidin
	Pi-Sigma	3.44996	LYS425	Petunidin
	Pi-Sigma	3.6684	ILE434	Petunidin
	Pi-Pi Stacked	5.44398	HIS357	Petunidin

	Pi-Pi T-shaped	5.53528	PHE429	Petunidin
	Alkyl	4.58386	Petunidin	VAL92
	Alkyl	4.65806	Petunidin	ILE434
P3G	Hydrogen Bond	2.87941	ARG128	P3G
	Hydrogen Bond	2.3503	LYS375	P3G
	Hydrogen Bond	2.87358	P3G	SER99
	Carbon Hydrogen Bond	3.73549	ARG128	P3G
	Pi-Cation	4.61441	LYS436	P3G
	Alkyl	4.47927	P3G	LYS436
	Pi-Alkyl	5.49258	P3G	LYS125
	Pi-Alkyl	4.34115	P3G	LYS436
	Pi-Alkyl	5.19298	P3G	ALA124
	Pi-Alkyl	4.74213	P3G	LYS125
	Pi-Alkyl	5.07534	P3G	LYS436
	Pi-Alkyl	5.00024	P3G	ARG437
	Pi-Alkyl	4.13541	P3G	LYS436
Peonidin	Hydrogen Bond	2.94428	ARG129	Peonidin
	Carbon Hydrogen Bond	3.44385	Peonidin	GLU96
	Pi-Donor Hydrogen Bond	2.9577	ARG128	Peonidin
	Alkyl	4.47099	Peonidin	LYS436
	Pi-Alkyl	4.29613	Peonidin	ARG128
	Pi-Alkyl	5.48054	Peonidin	ARG128
	Pi-Alkyl	5.11387	Peonidin	ILE132
	Pi-Alkyl	5.27203	Peonidin	ALA124
	Pi-Alkyl	3.97538	Peonidin	LYS125
PGA	Hydrogen Bond	2.3486	ASN297	PGA
	Hydrogen Bond	1.88965	PGA	ASN297
	Pi-Donor Hydrogen Bond	2.59293	PGA	HEM
	Pi-Pi T-shaped	4.77398	HEM	PGA
	Pi-Alkyl	4.57003	PGA	VAL117
	Pi-Alkyl	5.1465	PGA	LEU370
PCA	Hydrogen Bond	2.50237	ASN297	PCA
	Pi-Alkyl	4.23037	PCA	VAL117
	Pi-Alkyl	5.42791	PCA	LEU370
NNK	Pi-Pi T-shaped	4.97625	PHE107	NNK
	Pi-Alkyl	4.42944	NNK	VAL117
	Pi-Alkyl	4.72705	NNK	ILE300

NAT	Hydrogen Bond	2.73702	THR305	NAT
	Carbon Hydrogen Bond	3.44557	GLY301	NAT
	Pi-Pi T-shaped	5.64592	PHE209	NAT
	Alkyl	4.61665	VAL117	NAT
	Alkyl	5.10134	ILE300	NAT
	Pi-Alkyl	4.42335	PHE107	NAT
	Pi-Alkyl	5.14917	NAT	ILE366
Nicotine	Pi-Sigma	3.61671	Nicotine	PHE107
	Alkyl	4.4533	VAL117	Nicotine
	Alkyl	4.8371	ILE300	Nicotine
Coumarin	Hydrogen Bond	2.43742	ASN297	Coumarin
	Carbon Hydrogen Bond	3.51584	ASN297	Coumarin
	Pi-Pi T-shaped	5.12945	HEM	Coumarin
	Pi-Pi T-shaped	4.90209	HEM	Coumarin
	Pi-Pi T-shaped	5.24901	HEM	Coumarin
	Pi-Alkyl	4.45808	Coumarin	VAL117
	Pi-Alkyl	5.38546	Coumarin	LEU370
8-MOP	Pi-Sigma	3.95344	ILE366	8-MOP
	Pi-Pi Stacked	4.16518	PHE107	8-MOP
	Pi-Pi T-shaped	5.38689	PHE480	8-MOP
	Alkyl	5.44111	8-MOP	ILE300
	Pi-Alkyl	4.15422	PHE209	8-MOP
	Pi-Alkyl	5.35117	8-MOP	LEU370
	Pi-Alkyl	5.07911	8-MOP	VAL117
	Pi-Alkyl	5.06033	8-MOP	ILE300
	Pi-Alkyl	5.24387	8-MOP	LEU370
Cyp2a5				
C3G	Hydrogen Bond	3.0746	THR305	C3G
	Hydrogen Bond	1.93817	C3G	TYR438
	Pi-Cation	3.91323	HEM1:FE	C3G
	Pi-Sigma	3.58986	ALA301	C3G
	Pi-Pi Stacked	5.18544	PHE300	C3G
	Pi-Pi T-shaped	4.24422	HEM	C3G
	Pi-Alkyl	4.48417	C3G	VAL117
	Pi-Alkyl	4.17582	C3G	ALA301
	Pi-Alkyl	4.46289	C3G	VAL117
	Pi-Alkyl	4.61619	C3G	CYS439
Cyanidin	Pi-Sigma	3.82077	ALA301	Cyanidin

	Pi-Pi T-shaped	5.01725	PHE107	Cyanidin
	Pi-Alkyl	4.31127	Cyanidin	VAL117
	Pi-Alkyl	4.81457	Cyanidin	LEU370
	Pi-Alkyl	4.81722	Cyanidin	VAL117
	Pi-Alkyl	4.31876	Cyanidin	ALA301
	Pi-Alkyl	4.3841	Cyanidin	VAL117
	Pi-Alkyl	4.51468	Cyanidin	CYS439
Pt3G	Hydrogen Bond	3.02311	THR305	Pt3G
	Hydrogen Bond	3.03646	PHE440	Pt3G
	Hydrogen Bond	3.06245	Pt3G	PRO431
	Hydrogen Bond	2.59222	Pt3G	TYR438
	Carbon Hydrogen Bond	3.23707	CYS439	Pt3G
	Carbon Hydrogen Bond	3.26644	Pt3G	THR305
	Pi-Cation	3.84871	HEM1:FE	Pt3G
	Pi-Sigma	3.61061	ALA301	Pt3G
	Pi-Lone Pair	2.67346	Pt3G	HEM
	Pi-Pi Stacked	5.14217	PHE300	Pt3G
	Pi-Pi T-shaped	5.04035	PHE107	Pt3G
	Pi-Pi T-shaped	4.21703	HEM	Pt3G
	Pi-Alkyl	4.37109	Pt3G	A:VAL117
	Pi-Alkyl	5.42686	Pt3G	ALA301
	Pi-Alkyl	4.59254	Pt3G	CYS439
	Pi-Alkyl	3.23023	Pt3G	HEM
Petunidin	Hydrogen Bond	3.16309	PHE440	Petunidin
	Carbon Hydrogen Bond	3.24113	CYS439	Petunidin
	Pi-Cation	3.88501	HEM:FE	Petunidin
	Pi-Donor Hydrogen Bond	3.35255	Petunidin	PHE300
	Pi-Sigma	3.78507	ALA301	Petunidin
	Pi-Sigma	3.66699	Petunidin	PHE440
	Pi-Pi Stacked	5.21982	PHE300	Petunidin
	Pi-Pi T-shaped	4.98835	PHE107	Petunidin
	Pi-Pi T-shaped	5.26557	PHE118	Petunidin
	Pi-Pi T-shaped	4.33486	HEM	Petunidin
	Alkyl	2.49332	HEM1	Petunidin
	Alkyl	4.58514	Petunidin	CYS439
	Pi-Alkyl	3.53893	HEM	Petunidin
	Pi-Alkyl	4.31692	Petunidin	ALA301
	Pi-Alkyl	4.44658	Petunidin	VAL117
	Pi-Alkyl	4.61947	Petunidin	CYS439
	Pi-Alkyl	3.26749	Petunidin	HEM

P3G	Hydrogen Bond	3.04479	THR305	P3G
	Hydrogen Bond	3.18371	PHE440	P3G
	Hydrogen Bond	1.91415	P3G	ALA301
	Hydrogen Bond	2.67786	P3G	THR305
	Hydrogen Bond	2.94088	P3G	PRO431
	Carbon Hydrogen Bond	3.28835	CYS439	P3G
	Carbon Hydrogen Bond	3.2539	P3G	THR305
	Pi-Cation	3.86421	HEM:FE	P3G
	Pi-Sigma	3.61018	ALA301	P3G
	Pi-Pi Stacked	5.13458	PHE300	P3G
	Pi-Pi T-shaped	4.22247	HEM	P3G
	Pi-Alkyl	4.40519	P3G	VAL117
	Pi-Alkyl	5.43061	P3G	ALA301
	Pi-Alkyl	4.61131	P3G	CYS439
	Pi-Alkyl	3.23324	P3G	HEM1
Peonidin	Hydrogen Bond	3.13826	PHE440	Peonidin
	Hydrogen Bond	2.0478	Peonidin	TYR438
	Pi-Sigma	3.80289	ALA301	Peonidin
	Pi-Sigma	3.63773	Peonidin	PHE440
	Pi-Pi Stacked	5.22459	PHE300	Peonidin
	Pi-Pi T-shaped	4.98415	PHE107	Peonidin
	Pi-Pi T-shaped	5.28598	PHE118	Peonidin
	Pi-Pi T-shaped	4.20571	HEM	Peonidin
	Alkyl	2.47853	HEM	Peonidin
	Alkyl	4.56895	Peonidin	CYS439
	Pi-Alkyl	3.57314	HEM	Peonidin
	Pi-Alkyl	4.38218	Peonidin	VAL117
	Pi-Alkyl	4.32508	Peonidin	ALA301
	Pi-Alkyl	4.434	Peonidin	VAL117
	Pi-Alkyl	4.63549	Peonidin	CYS439
	Pi-Alkyl	3.28323	Peonidin	HEM
PGA	Hydrogen Bond	3.02427	ARG101	PGA
	Hydrogen Bond	2.93585	ARG372	PGA
	Hydrogen Bond	2.27145	PGA	ARG437
	Amide-Pi Stacked	5.34876	TYR438	PGA
	Pi-Alkyl	4.84792	PGA	LEU370
	Pi-Alkyl	5.10872	PGA	CYS439
PCA	Hydrogen Bond	3.02067	ARG101	PCA
	Hydrogen Bond	3.12102	LEU370	PCA
	Hydrogen Bond	2.10997	PCA	PRO431

	Hydrogen Bond	2.55591	PCA	SER433
	Amide-Pi Stacked	4.81024	PHE432	PCA
	Pi-Alkyl	4.76567	PCA	LEU370
NNK	Hydrogen Bond	2.90284	ARG128	NNK
	Hydrogen Bond	3.0511	PHE440	NNK
	Hydrogen Bond	3.12765	GLY441	NNK
	Pi-Alkyl	4.1981	NNK	ALA301
	Pi-Alkyl	3.68798	NNK	HEM
NAT	Hydrogen Bond	3.38026	ASN297	NAT
	Carbon Hydrogen Bond	3.26159	ASN297	NAT
	Pi-Pi Stacked	4.72684	PHE300	NAT
	Pi-Pi T-shaped	4.8294	PHE107	NAT
	Pi-Pi T-shaped	5.01011	PHE118	NAT
	Alkyl	5.32142	ALA301	NAT
	Alkyl	5.38006	ILE366	NAT
	Alkyl	4.80634	LEU370	NAT
	Pi-Alkyl	5.34599	PHE480	NAT
	Pi-Alkyl	4.92182	HEM	NAT
	Pi-Alkyl	4.85945	NAT	VAL117
	Pi-Alkyl	4.6271	NAT	ALA301
Nicotine	Pi-Pi Stacked	4.39887	PHE300	Nicotine
	Pi-Pi T-shaped	4.76129	PHE107	Nicotine
	Pi-Pi T-shaped	5.21533	PHE118	Nicotine
	Pi-Alkyl	5.41022	PHE107	Nicotine
	Pi-Alkyl	5.25087	PHE209	Nicotine
	Pi-Alkyl	5.22124	PHE480	Nicotine
	Pi-Alkyl	5.17103	Nicotine	VAL117
	Pi-Alkyl	4.72528	Nicotine	ALA301
Coumarin	Pi-Pi Stacked	4.70562	PHE300	Coumarin
	Pi-Pi T-shaped	4.78322	PHE107	Coumarin
	Pi-Pi T-shaped	5.10711	PHE118	Coumarin
	Pi-Alkyl	4.44768	Coumarin	ALA301
	Pi-Alkyl	4.94814	Coumarin	VAL117
	Pi-Alkyl	4.52058	Coumarin	ALA301
8-MOP	Hydrogen Bond	3.22915	ASN297	8-MOP
	Carbon Hydrogen Bond	3.2662	ASN297	8-MOP
	Pi-Sigma	3.75161	LEU370	8-MOP
	Pi-Lone Pair	2.58261	8-MOP	HEM

	Pi-Pi Stacked	5.75185	PHE300	8-MOP
	Pi-Pi T-shaped	5.17338	PHE107	8-MOP
	Pi-Pi T-shaped	4.92811	PHE118	8-MOP
	Pi-Pi T-shaped	4.93202	HEM	8-MOP
	Alkyl	3.6102	ALA301	8-MOP
	Alkyl	4.61527	8-MOP	VAL117
	Pi-Alkyl	4.56174	HEM	8-MOP
	Pi-Alkyl	4.74614	8-MOP	ILE366
	Pi-Alkyl	4.44734	8-MOP	VAL117
	Pi-Alkyl	4.4091	8-MOP	ALA301
	Pi-Alkyl	4.73163	8-MOP	LEU370

Supplementary Table S3. The results of the structure validation in SWISS-Model server for predicted AlphaFold 3 cyp2a5 in reference to CYP2A13

**Parameter**	**Score**
IDDT	0.90
TM-score	0.99
RMSD	0.61
MolProbity score	1.16
Clash score	3.7
Ramachandran favoured	98.17%
Ramachandran outliers	0.00%
Rotamer outliers	0.46%
C-beta deviations	0
Bad bond	0.02%
Bad angles	0.00%
QMEAN	0.89 ± 0.05

Supplementary Figures

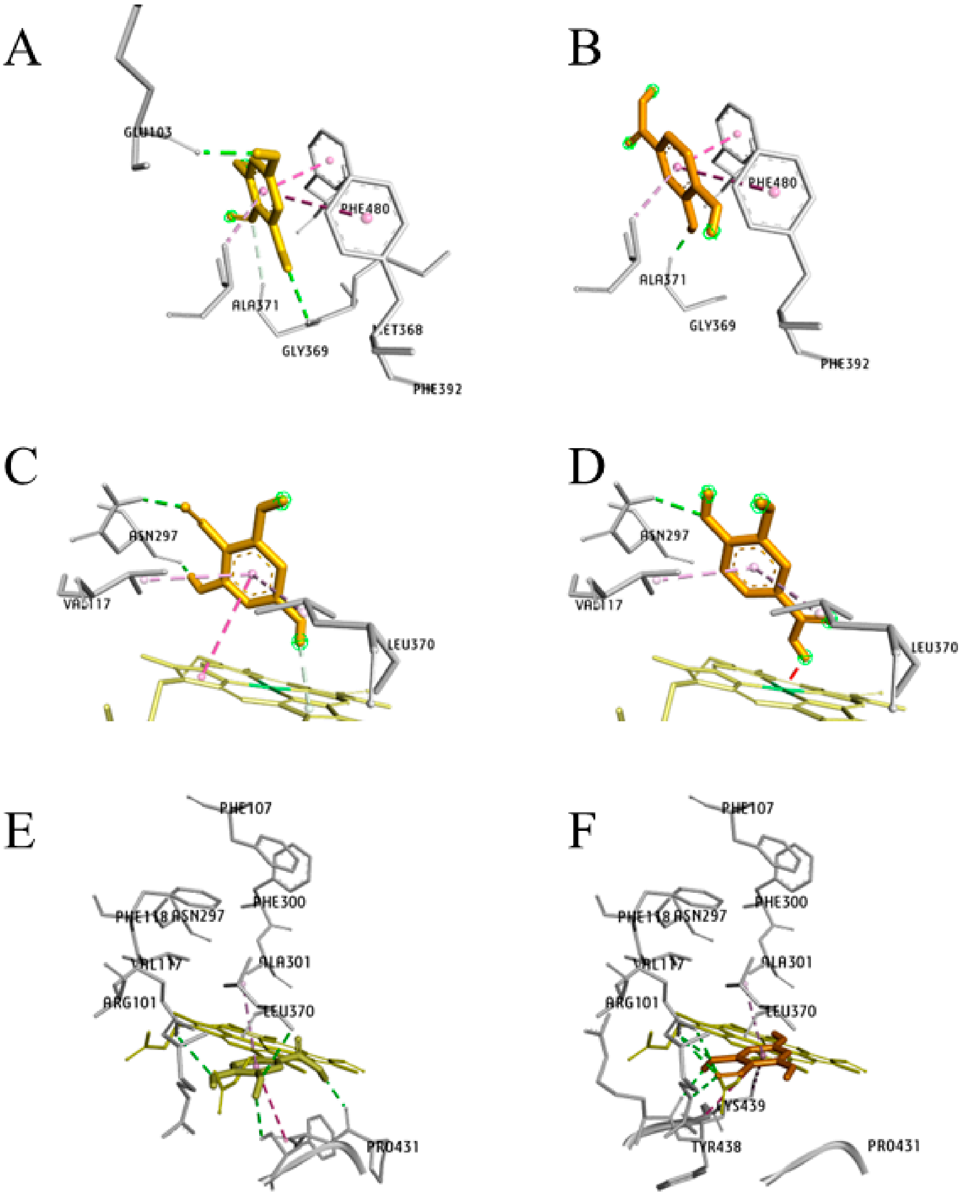

Supplementary Figure S1.
The high-affinity PCA and PGA, two primary phenolic metabolites of C3G, are shown in the three-dimensional plots. A and B) The interaction of PCA and PGA with the active site of CYP2A13 enzyme. C and D) The interaction of PCA and PGA with the active site of CYP2A6 enzyme. E and F) The interaction of PCA and PGA with the active site of cyp2A5 enzyme. Colour coding of ligand-amino acid interactions, green, conventional hydrogen bonds; pink, Pi bonds; purple, Sigma bonds; red, donor-donor bonds. Figures A – F are generated using BIOVIA Discovery Studio Visualizer V21.1.0.20198. PCA, protocatechuic acid; PGA, phloroglucinaldehyde.

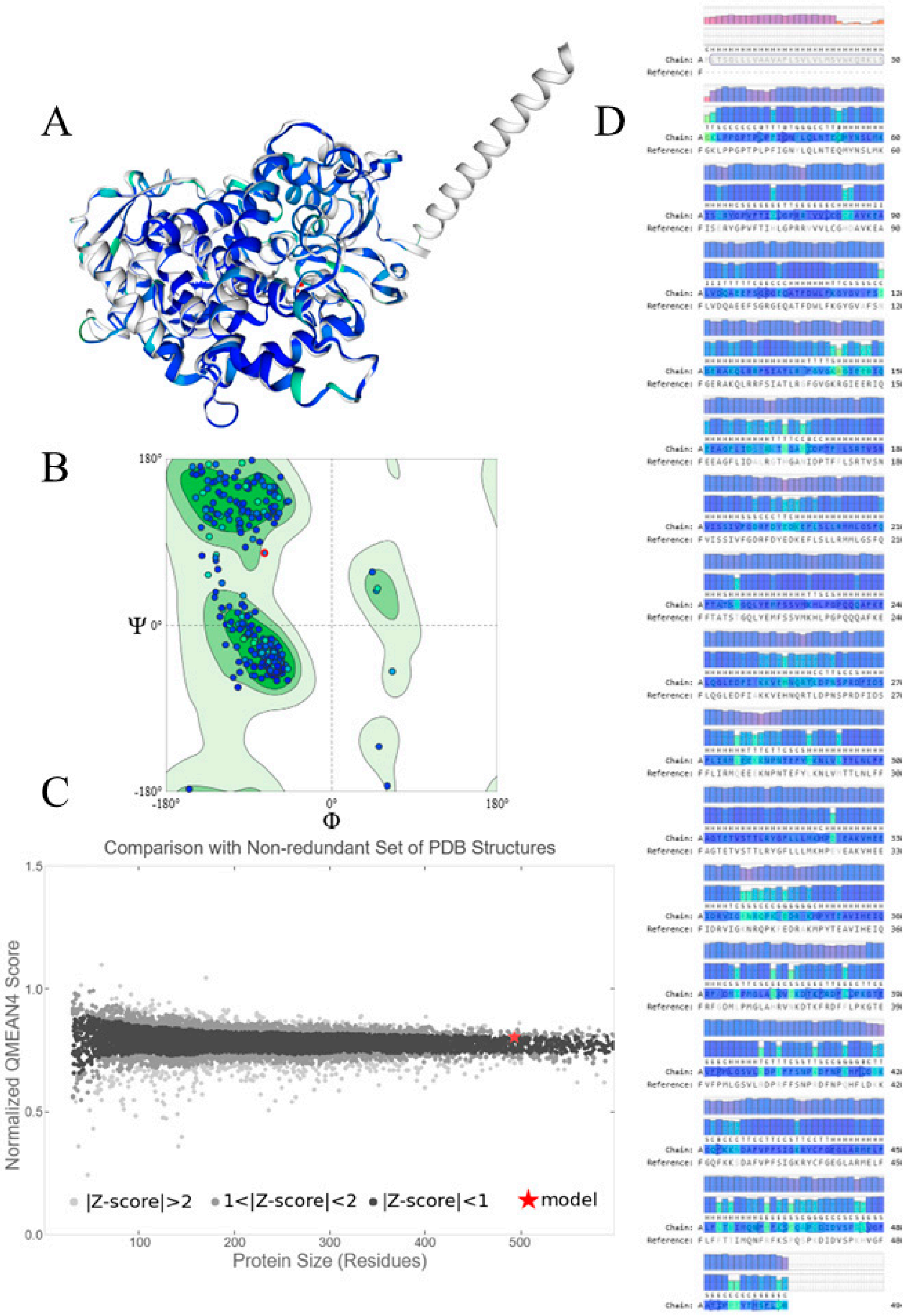

Supplementary Figure S2. The quality assessment of heme-bound cyp2a5 AlphaFold 3 prediction with its homology CYP2A13 in SWISS-MODEL structure assessment and modelling server. A) Alignment of cyp2a5 model (blue) with its homologuos CYP2A13 (grey) B) Ramachandran plot of cyp2a5. C) comparison of the cyp2a5 model (red star) with non-redundant set of PDB structures. D) Alignment of the amino acid residues.
